# Premeiotic chromatin states orchestrate gene expression during male gametogenesis in rice

**DOI:** 10.1186/s13059-026-04129-4

**Published:** 2026-06-12

**Authors:** Bo Zhu, Feng Zhao, Qian Liu, Chenxi Pu, Miaomiao Ye, Pengxiang Bai, Emmanuel Guiderdoni, Yu Zhao, Dao-Xiu Zhou

**Affiliations:** 1 National Key Laboratory of Crop Genetic Improvement, Hubei Hongshan Laboratory, Huazhong Agricultural University, Wuhan, 430070 China; 2 CIRAD, AGAP Institute, Avenue Agropolis, BP 5035, Montpellier, 34398 France; 3 University of Montpellier, CIRAD-INRAE-Institut Agro, University of Montpellier, Montpellier, France; 4 Institute of Plant Science Paris-Saclay (IPS2), CNRS, INRAE, Université Paris-Saclay, Orsay, 91405 France

**Keywords:** Rice male gametogenesis, Meiocyte, Microspore, Sperm, Chromatin remodeling, Bivalent histone methylation, Gene body DNA methylation (gbM), Haploid, Pollen selection

## Abstract

**Background:**

Male meiocyte specification from somatic tissue and male germ line development require extensive epigenetic reconfiguration. However, how the extent and the timing of the reconfiguration required for gene expression during the male lineage development remains unclear in rice.

**Results:**

Here, we integrate cell type-specific transcriptomic and epigenomic profiling across the rice male germline, from meiocytes to sperm cells. We show that gene expression programs in male gametic cells are largely prefigured by chromatin states established in meiocytes. In particular, H3K4me3 deposition initiated during meiosis is broadly maintained throughout development, whereas H3K27me3 is progressively reduced, contributing to stage-specific gene activation. DNA methylation patterns are largely stable, except for a transient reduction of CHH methylation in early microspores, accompanied by increased chromatin accessibility and enhanced H3K4me3. Notably, genes activated during microspore development are depleted of gene body DNA methylation (gbM), while gbM-enriched genes are preferentially repressed in sperm cells. Functional analysis of H3K4 methyltransferases demonstrates that pre-establishment of H3K4me3 is required for proper microspore gene expression and development.

**Conclusion:**

Our findings reveal that a premeiotic chromatin blueprint is maintained to instruct transcriptional programs during male gametogenesis. This epigenetic configuration preferentially activates non-constitutive genes lacking gbM, suggesting a mechanism that may enhance regulatory flexibility and facilitate haploid selection. These results provide a conceptual framework for how chromatin state inheritance shapes gene expression and evolutionary dynamics in the plant male germline.

**Supplementary Information:**

The online version contains supplementary material available at 10.1186/s13059-026-04129-4.

## Background

The male gametophyte develops from pollen mother cells (or male meiocytes) through meiosis into microspores, which then undergo mitosis to give rise to binuclear microspores and ultimately mature into pollen grains containing two sperm cells and a vegetative nucleus [1]. Unlike animal primordial germ cells that are elaborated during early embryogenesis, plant germlines are developed from somatic cells at a late developmental stage [1, 2]. In addition, in mammals there is extensive epigenetic reprogramming such as the global erasure of DNA methylation in primordial germ line and in zygote after fertilization [3], while plant reproduction is accompanied only by a partial epigenetic reprograming process [4, 5], with local remodeling of DNA methylation in the gametes or zygote [6–10]. DNA methylation is a hallmark for transposable element (TE) and repetitive sequence silencing in flowering plants and vertebrates. In flowering plants, DNA cytosine methylation mainly occurs in the CG, CHG, and CHH (where H is A, C, or T) sequences. It is shown that Arabidopsis meiocyte CHH methylation is reinforced by siRNAs produced in the tapetum through the RNA-directed DNA methylation pathway, and the reinforced methylation state is maintained throughout the entire male gametophyte development [11], similar to what observed in sperm cells in which DNA methylation at TE loci is reinforced by siRNAs produced in the vegetative nucleus [12, 13]. Although DNA methylation generally induces gene silencing, the function of gene body methylation (gbM) that occurs only in the CG context at exons of constitutively expressed genes has been extensively debated [14]. gbM is common in animals and is nearly ubiquitous in flowering plants [15].

The specification of male meiocytes from somatic tissue marks the somatic-to-reproductive cell fate transition. It has been shown that Arabidopsis male meiocyte differentiation is accompanied by large-scale changes in chromatin organization including chromatin decondensation and quantitative changes in histone modifications [16]. Epigenomic profiling in the rice male meiocytes at prophase I revealed that abundance of H3K4me3 (an active mark) and H3K27me3 (a repressive mark) was correlated and anti-correlated with gene expression levels, respectively [17]. Several studies indicate that histone modification is extensively reconfigured in pollen. For instance, H3K27me3 is found to be largely depleted from sperm cells in Arabidopsis [18]. The regions lacking this mark are significantly enriched for H3K4me3, a modification associated with gene activation that promotes sperm cell development. Notably, many of the H3K4me3-marked genes are not expressed in the sperm cells but are rapidly activated following fertilization [18]. In the endosperm, paternally expressed imprinted genes display high gbM levels on the paternal alleles, while the maternal alleles are densely marked by H3K27me3 [19]. Collectively, these findings suggest that H3K27me3 reconfiguration in sperm cells may establish a prerequisite to instruct gene expression in the early embryo and endosperm. In addition, Arabidopsis sperm cells have apparent chromatin bivalent loci established likely by the acquisition of H3K27me3 or H3K4me3 at pre-existing H3K4me3 or H3K27me3 regions, respectively [20]. By contrast, maintenance of high H3K27me3 levels is essential for vegetative cell fate commitment in Arabidopsis male gametophytes [21]. Recent results in tomato plants suggest that the asymmetric division of the microspore significantly reshapes the genome-wide distribution of H3K4me3 in sperm and vegetative cells [22]. However, these data were obtained mainly at one or few stages/cell types of male gametogenesis. Studies on the dynamic changes of epigenomes and transcriptomes at the different stages/cells during male gametogenesis are needed to underpin epigenetic mechanism of male gametophyte gene expression and development.

Here, we obtained datasets of cell type-specific transcriptomes and epigenomes of the rice male germ lineage isolated at the different developmental stages. Our results pinpoint to a premeiotic set-up of a chromatin state that instructs cell-type specific gene expression during male gametogenesis and uncover a specific chromatin signature for male gamete gene expression which might have implication in haploid selection.

## Results

### Dynamic transcriptomic changes during male germline development

**Fig. 1:**
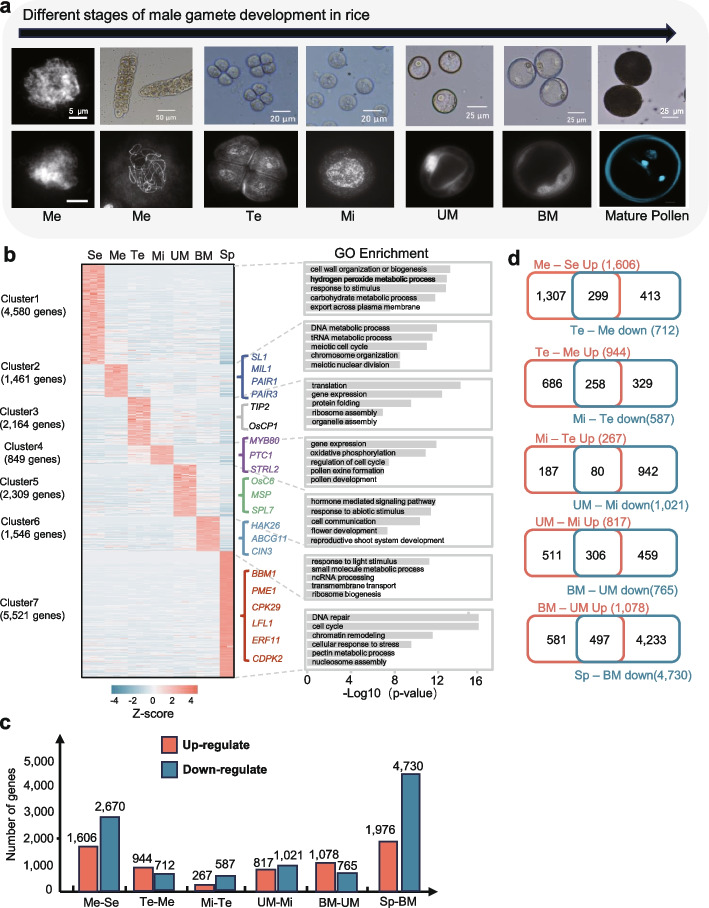
Transcriptomic dynamics during male gamete development. **a** Cell morphology (upper part) and DAPI staining (lower part) during pollen development. Se, seedling; Me, meiocyte (at meiosis prophase I, which mainly includes the leptotene, zygotene, and pachytene stages); Te, tetrad; Mi, or early undifferentiated uninucleate microspores (UNM); UM, or vacuolated UNM or polarized UNM; BM, bicellular microspore or bicellular pollen (BCP); Sp, sperm. **b** Clustering and gene ontology (GO) enrichment of transcripts with the highest levels in each indicated cell type, with FPKM > 1; fold change > 1.5; *p*-value < 0.01; higher than the other cell types. Three replicates are shown. A total of 18,430 genes were analyzed. **c** Numbers of differentially expressed genes between adjacent developmental stages (fold change > 2; adjusted *p*-value < 0.01; with FPKM > 3 for upregulated genes and FPKM < 1 for downregulated genes). **d** Overlaps between the downregulated genes in the male cells (relative to the previous stage) with the upregulated ones in the previous stage or cell type

In rice, all meiocytes in an anther progress through meiosis in unison and all anthers within a spikelet are at the same developmental stage [23]. Based on cell morphology and DAPI staining, rice male germ cells at the different stages could be distinguished in developing anthers. We individually isolated meiocytes (Me, at meiosis prophase I, which mainly includes the leptotene, zygotene, and pachytene stages), tetrad (Te), undifferentiated microspores (Mi, corresponding to early undifferentiated uninucleate microspores, or UNM); unicellular microscopes (UM, corresponding to vacuolated UNM or polarized UNM), and bicellular microspores (BM, corresponding to bicellular pollen, or BCP) with thick cell wall, more prominent large vacuoles, and the presence of two nuclei (Fig. 1a, Additional file 1: Fig. S1a). Sperm (Sp) were isolated from mature pollen grains using a previously described method [24] with modifications to improve purity (see Methods) (Additional file 1: Fig. S1b).

About 100–200 Me, Te, Mi, UM and BM and about 1,000 Sp were pooled for single cell-type RNA-seq (Additional file 2: Table S1). Three replicates (except two for WT sperm) were analyzed with a mean of 19,901 transcripts (with Fragments Per Kilobase of transcript per Million mapped reads, FPKM, > 1) detected per sample (Additional file 2: Table S1). The replicates showed high reproducibility (Person > 0.99, Additional file 1: Fig. S2a). Principal component analysis (PCA) revealed a transcriptomic trajectory reflecting the developmental chronology from Me to BM (Additional file 1: Fig. S2b). Of note, the position of Mi transcriptome was close to that of Te, attesting the early developmental stage of Mi. The Sp transcriptome was largely distal from those of the microspores in the PCA. Clustering of transcript abundance revealed 849 to 5,521 genes with the highest expression in each male cell types (Fig. 1b). Large numbers of differentially expressed genes detected in Me-Se and Sp-BM were consistent with previous results showing a large shift of gene expression occurred during early meiosis and in sperm [25, 26] (Fig. 1c). Genes highly expressed in Me were enriched for meiosis function (Fig. 1b), including multiple meiotic marker genes whose expression dramatically dropped in Te (Additional file 1: Fig. S3). Genes expressed in the Te stage were enriched for translation and gene expression functions, while those in Mi were enriched for gene expression, cell cycle and pollen development (Fig. 1b). However, in more differentiated UM the highly expressed genes were related to hormone signaling, stress, and cell communications (Fig. 1b). In BM that contains a generative cell and a vegetative cell, the highly expressed genes included those involved in ncRNA processing that is shown to be active in the vegetative cell [12, 13], and ribosome biogenesis that is known to be shut down in Me during meiosis [25] (Fig. 1b). In Sp, the expression of genes involved in cell cycle, chromatin structure was the highest (Fig. 1b). In addition, a dynamic change of transcript levels was observed during microspore differentiation. For instance, 258 of the 944 genes upregulated in Te became downregulated in Mi (Fig. 1c, d), and 306 of the 817 genes upregulated in UM became downregulated in BM, in which the other 1,078 genes were upregulated (Fig. 1c, d). Most of the up- and downregulated genes displayed variations in expression levels at different stages (Additional file 1: Fig. S2c, d). Collectively the dataset provides a time course of gene expression during rice male germline development.

### Chromatin dynamics during male germline development

To study the chromatin basis of gene expression during male gametogenesis, we analyzed genome-wide profiles of histone H3K4me3, H3K9me2, H3K27me3, H3K36me3, and H3 (as control), open chromatin, and DNA methylation in the different male cell types (Additional file 1: Fig. S1c, Additional file 1: Fig. S4d, Additional file 2: Tables S2, S3, S4). CUT&Tag analysis of histone methylations identified 27,636 to 35,336 peaks of H3K4me3, 8,456 to 11,141 peaks of H3K9me2, 6,879 to 14,456 peaks of H3K27me3, and 13,345to 18,987 peaks of H3K36me3 in the different male cell types (Additional file 2: Table S2). The histone methylation CUT&Tag data showed a high reproducibility between the replicates (Pearson correlation coefficient *R* > 0.85) (Additional file 1: Fig. S4a), but not with the H3 control (Additional file 1: Fig. S4b), indicating a high specificity of the antibodies. H3K4me3 CUT&Tag and chromatin immunoprecipitation (ChIP)-seq peaks from Se were similar (*R* > 0.8) (Additional file 1: Fig. S4c), suggesting no technical bias.

PCA analysis of DNA methylomes revealed a good repeatability of the samples of same cell types with clear differences detected between the cell types (Additional file 1: Fig. S5a). Compared to other male cell types, Mi showed clear DNA methylation differences especially at CHG and CHH sequences (Additional file 1: Fig. S5b, c). CG methylation (mCG) is relatively stable, with some losses (mCG hypo DMRs) detected in Me relative to somatic cells, which were basically maintained at later stages (Additional file 1: Fig. S5d). By contrast, non-CG methylations showed more dynamically changed during male germline development, with clearly decreases of both mCHG and mCHH in Mi, which were however restored in UM (Additional file 1: Fig. S5b-e). However, the overall mCHH level increased in Me, which was enhanced in UM and Sp cells (Additional file 1: Fig. S5b-e). The drop of mCHH in Mi might be a consequence of dilution by meiotic cell division, as, unlike mCG and mCHG which are maintained during S and M phases of cell cycle, mCHH is shown to be gradually maintained after cell division [32]. However, a possible active demethylation process is not ruled out. The DNA methyltransferase OsCMT3b that is specifically expressed in rice male gametes [8], may contribute to the maintenance or enhancement of non-CG methylation in UM.

Because the non-CG DMRs were predominantly located at TEs (Additional file 1: Fig. S5d), we analyzed the associations between DNA methylation and TE expression in the male cells. The overall TE expression levels, normalized by RPM (Reads Per Million mapped reads), were the highest in UM (Additional file 1: Fig. S6a, Additional file 2: Table S5). In total, 8,510 TE were found to be expressed in UM compared to 2,457 in Me, and 2,443 in Mi (Additional file 1: Fig. S6b). Notably, the number of expressed LTR/Gypsy increased sharply in UM (Additional file 1: Fig. S6c). Surprisingly, the UM-expressed TEs displayed higher than the average mCG and mCHG levels of all TEs at all stages examined, while the TEs expressed in Me and Mi showed lower methylation levels. No clear change in histone methylations was detected in the expressed TEs (Additional file 1: Fig. S6d). This suggests that TE expression in UM is independent of DNA methylation or the histone modifications examined.

**Fig. 2:**
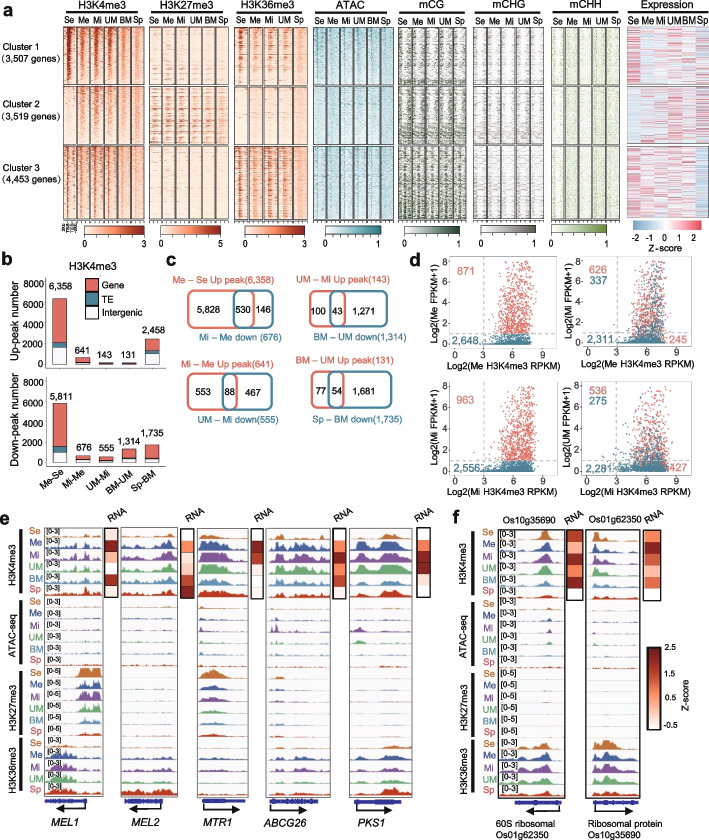
H3K4me3 is reconfigured in meiocyte and maintained during male gamete development. **a** Heatmaps of gene H3K4me3 levels in the indicated cell types. Cluster 1: genes with loss of broad H3K4me3 in body region in Me and the haploid male cells; Cluster 2: genes with gain of broad H3K4me3 in Me and the haploid male cells. Clusters 3: Genes with constant sharp H3K4me3 near the transcription start sites (TSS) in Se, Me, Mi and UM, but with reduced H3K4me3 levels in BM and Sp. H3K27me3, H3K36me3, ATAC-seq signal, DNA methylation (at CG, CHG, and CHH sites), and expression levels of the H3K4me3-marked genes are shown. Heatmaps in this study were generated using deepTools software, with BPM (Bins Per Million mapped reads) as the normalization method. **b** Numbers of differentially methylated peaks between adjacent developmental stages (fold change > 1.5; adjusted *p*-value < 0.01). **c** Overlaps between the downregulated peaks in the male cells (relative to the previous stage) with the upregulated ones in the previous stage or cell type. **d** Left panels: Scatter plots of expression levels (y-axis) in Me (upper part) or Mi (lower part) of cluster 2 genes that gained H3K4me3 (x-axis) in Me (upper part) or Mi (lower part). The orange dots represent expressed genes at the Me or Mi stage, while the blue dots represent silent genes. Right panels: expression changes between Me and Mi (upper part) or between Mi and UM (lower part) of cluster 2 genes that gained H3K4me3 in Me or Mi. In Mi vs Me, 337 and 245 genes were respectively up or down regulated, while 626 genes remain expressed. In UM vs Mi, 275 and 427 genes were up or downregulated, while 536 genes remain expressed. **e**,** f** Integrative Genomics Viewer (IGV) of representative marker genes from Cluster 2 (**e**) and Cluster 3 (**f**)

To investigate activity of H3K4me3-marked genes during male gametogenesis, we divided the marked genes into two categories: one with the mark spanning entire gene body and other with the mark specifically enriched at transcription start site (TSS). When both H3K4me3 and expression levels were considered, three clusters were identified. Cluster 1 genes (3,507) with broad H3K4me3 in gene body region in Se showed losses of the mark and were downregulated in the male cells (Fig. 2a, Additional file 1: Fig. S7a). These genes also showed decreases of H3K36me3 and were enriched for photosynthesis, metabolism and regulation of transcription (Additional file 1: Fig. S7a, b). Conversely, cluster 2 genes (3,519) gained broad H3K4me3 in Me and maintained the mark throughout the male germline development (Fig. 2a, b, c, Additional file 1: Fig. S7a, e). Among these genes, 871 were transcribed (FPKM > 1) in Me, of which, 626 maintained to be expressed in Mi where the other 337 genes become expressed (Fig. 2d). Similarly, from the 963 genes expressed in Mi, 536 remained to be expressed in UM where 275 additional genes started to express (Fig. 2d). These genes were also marked by H3K27me3, the level of which gradually decreased during the male germline development, which may contribute to the sequential expression of this group of H3K4me3-marked genes (Additional file 1: Fig. S7a). About 60% of cluster 2 genes showed upregulation in the male cells, while the remaining genes were constantly silent (Additional file 1: Fig. S7f). The silent genes had relatively higher H3K27me3 levels (Additional file 1: Fig. S7g), resulting in lower H3K4me3/H3K27me3 ratio that is suggested to affect gene expression in sperm [20]. The genes activated in male cells were mostly enriched for response to oxidative stress (Additional file 1: Fig. S7c), consistent with oxidative burst observed in pollen [27], stamen and anther development functions including known male gamete genes such as *MEL1* [28], *MEL2* [29], *MTR1* [30], *ABCG26* [31] and *PKS1* [32] (Fig. 2e). Loss of H3K27me3 appeared to be related to upregulation of *MEL*, *MTR1*, while gain of H3K4me3 or H3K36me3 was mostly related to upregulation of *ABCG26* and *PKS1* or *MEL2*, respectively. Cluster 3 genes (4,453) were marked by a sharp H3K4me3 signal at TSS and H3K36me3 in gene body in Se, Me and the microspores but showed decreases of the marks and downregulation of expression in Sp (Fig. 2a, Additional file 1: Fig. S7a). These genes were mainly enriched for translation functions, consistent with the shutdown of translational activity in Sp [33] (Fig. 2f, Additional file 1: Fig. S7d). Interestingly, genes in clusters 1 and 2 (with loss or gain of broad H3K4me3 respectively in Me and the haploid male cells) showed no or low gbM, while those in clusters 3 (with sharp H3K4me3 at TSS maintained in Me and the early haploid male cells but reduced in Sp) were enriched for gbM (Fig. 2a, Additional file 1: Fig. S7a).

**Fig. 3:**
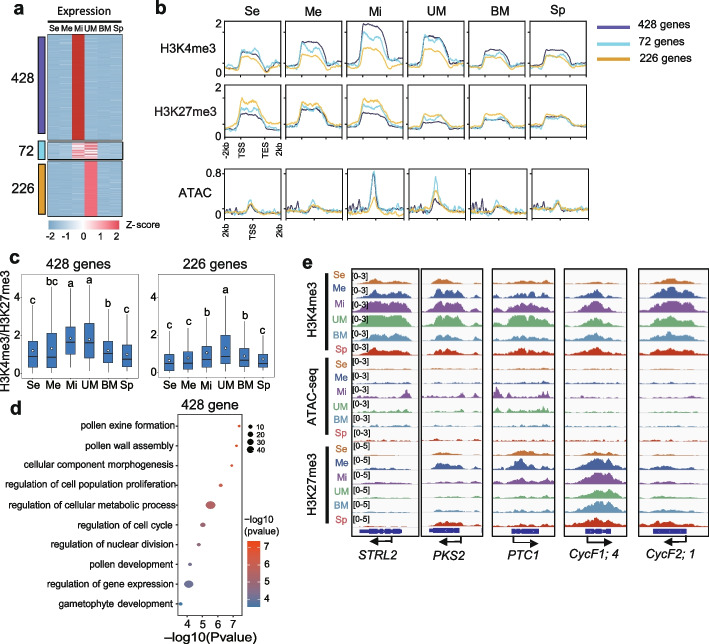
Chromatin signatures of genes specifically expressed in Mi and UM. **a** Chromatin modifications of Mi and/or UM –specific genes (FPKM > 3, SPM > 0.9) in Se, Me and the haploid male cells. **b** The metaplot of H3K4me3, H3K27me3, and ATAC levels for three categories of genes: Mi-specific expression (purple), Mi and UM-expressed (light blue), and UM-specific expression (orange). Metaplots in this study were generated using deepTools software, with BPM (Bins Per Million mapped reads) as the normalization method. **c** boxplot of H3K4me3/H3K27me3 ratios for the 428 and 226 genes in a. The average levels (white dots) and median values (black bars) are indicated. Significance was calculated using multiple comparison tests. Different lettering above the bars indicates significant differences (*p* < 0.01). **d** GO enrichment analysis of 428 genes. **e** Integrative Genomics Viewer (IGV) of representative marker genes from 428 genes

The genes that are specifically expressed in Mi showed the highest H3K4me3/H3K27me3 ratio and ATAC signals in Mi, while those specifically expressed in UM displayed the highest H3K4me3/H3K27me3 ratio and ATAC signal in UM (Fig. 3a-c), supporting the notion that the H3K4me3/H3K27me3 ratio is important for open chromatin and gene expression in male gametes [20]. These Mi-expressed genes are significantly enriched in pollen development and cell cycle pathways (Fig. 3d, e).

Analysis of H3K27me3 found that 615 genes (cluster 1) showed clear losses of the mark and expression in Me and the male gametic cells (Additional file 1: Fig. S8a). These genes were enriched for functions in floral development (Additional file 1: Fig. S8b), including known regulatory genes *MADS3* [34]*, MADS58* [35] and *PAP2* [36]*, SPW1* [37] (Additional file 1: Fig. S8c). By contrast, 1,627 genes (cluster 2) that were marked by broad H3K4me3 gained H3K27me3 in Me and Mi (Additional file 1: Fig. S8a). These genes showed reduced expression in male cells, especially in Me, likely due to the lowest ratio of H3K4me3/H3K27me3 observed in Me (Additional file 1: Fig. S8d, e). The overall H3K27me3 level was gradually lost during male gamete development (Additional file 1: Fig. S8f).

H3K36me3 occurs in gene body and is associated with gene expression. Enrichment of both H3K4me3 on the promoter and of H3K36me3 on the gene body represents a universal signature of gene activity [38]. Analysis of H3K36me3 revealed that a similar number of genes lost (cluster 1) or gained the mark (cluster 2) in the male cells (Additional file 1: Fig. S9a, b). These genes also displayed concomitant changes of H3K4me3 (either in body or at TSS), but without alteration in gbM (Additional file 1: Fig. S9a, b). However, the overall H3K36me3 levels were lower in Me and Sp, which may be related to the more condensed chromatin state at these stages (Additional file 1: Fig. S9c).

H3K9me2 is a hallmark of heterochromatin, associated with DNA methylation and gene silencing. We found that H3K9me2 was stably maintained from somatic cells to male cells, although a slight decrease could be observed in Sp (Additional file 1: Fig. S10a, b). Relatively few H3K9me2 differential peaks were detected, which were almost exclusively annotated to TEs (Additional file 1: Fig. S10c).

**Fig. 4:**
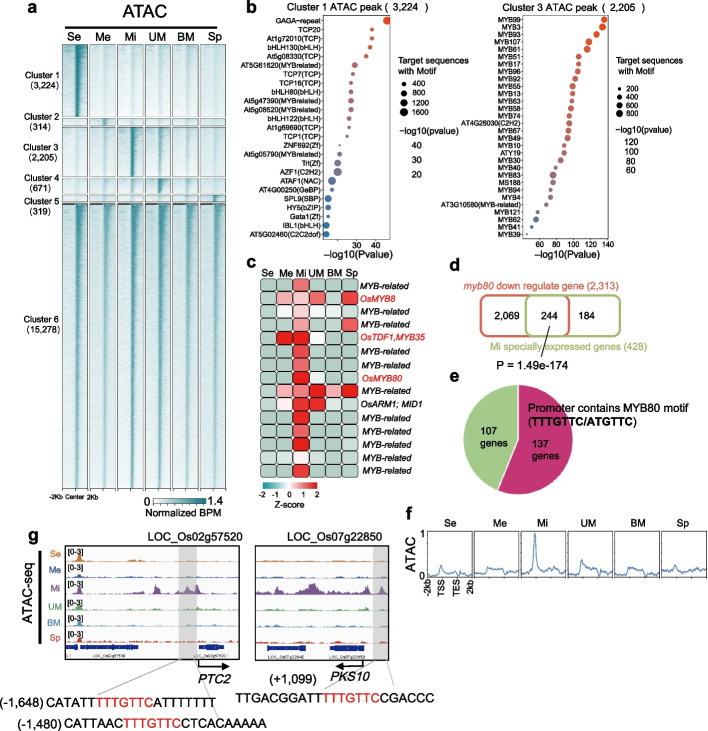
MYB transcription factor (MYB80) in triggering Mi gene expression. **a** Clustering by heatmaps of ATAC signals detected in Se, Me and the haploid male cells. Clusters 1–5: cell type- or stage-specific ATAC-seq signals; Cluster 6: ATAC-seq signals detected in all developmental stages. **b** Enrichment of transcription factors binding sites in Se and Mi cell type-specific ATAC peaks (Clusters 1 and 3 in Fig. a, Using predictions of transcription factor binding sites in Arabidopsis thaliana). **c** Heatmap of male cell type-specific expression of MYB transcription factor genes. Genes highlighted in red are previously characterized to function in male gamete development. **d** Overlap between Mi-specific expressed genes and downregulated genes in rice myb80 mutant anthers at stage 9 (Mi period; reported by Pan et al. [40]). The *P*-values were calculated by hypergeometric tests. **e** Among the 244 genes in d, 137 contain MYB80-binding motif in their promoter regions. **f** The metaplot of ATAC signal levels of the 137 genes shown in (**e**). **g** IGV of ATAC signals in two of the 137 genes in the different cell types. The MYB-binding sites with ATAC signals in Mi cells are shaded

ATAC sites are open chromatin regions accessible for gene regulation. ATAC profiling found that 3,224 open chromatin sites detected in Se were lost in Me and the haploid male cells (Cluster 1), while a similar number of sites (clusters 2–5) sequentially gained the ATAC signal during male germline development, consistent with previous results showing extensive reconfiguration of chromatin accessibility during microspore to pollen development in Arabidopsis [39] (Fig. 4a). The male cell type-specific ATAC clusters showed relatively higher expression levels compared to the other cell types (Additional file 1: Fig. S11a). Genes gained ATAC signals in Mi were enriched for pollen development-related functions (Additional file 1: Fig. S11b, c). Genes with variation of ATAC signals were enriched for H3K4me3 and H3K36me3 (Additional file 1: Fig. S11d), or reduced H3K27me3 (Additional file 1: Fig. S11e).

The Mi-specific ATAC sites (Fig. 4a, cluster 3) were highly enriched for MYB transcription factors binding sequences (Fig. 4b). Consistently, several rice *MYB* genes were specifically and highly expressed in the microspores, particularly in Mi (Fig. 4c). Among them, *MYB80* was shown to be required for microspore development [40]. We found that 244 of the 428 Mi-specific genes were downregulated in rice *myb80* mutant anthers (at stage 9, corresponding to the Mi stage) (Fig. 4d). Among them, 137 genes contain the MYB80-binding motif in their promoter regions and are enriched for ATAC signals at the Mi stage [40] (Fig. 4e-g). The analysis suggested that MYB80 might play a role in Mi gene expression in rice.

### Chromatin basis of male cell type-specific gene expression

**Fig. 5:**
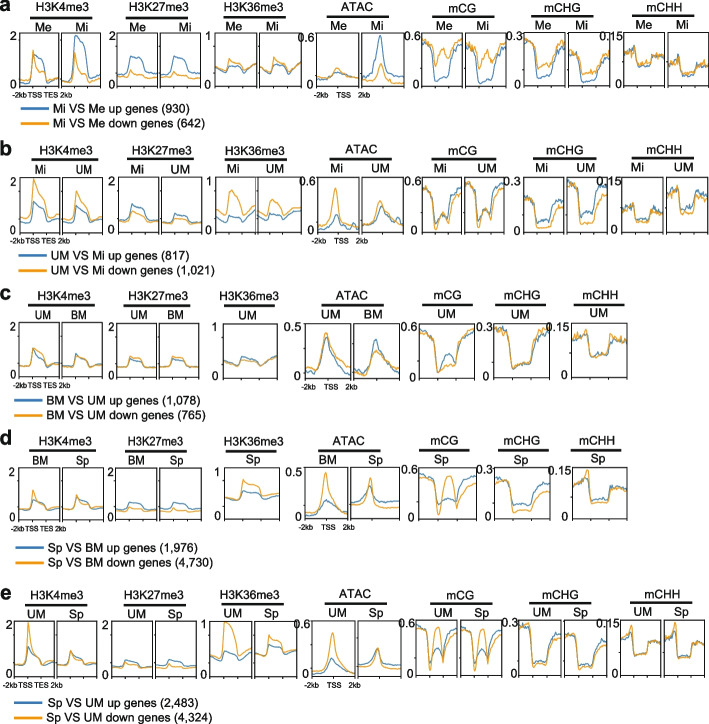
Chromatin states for male cell type-specific gene expression. Metaplots of chromatin modification levels of the differentially expressed genes in Me and the haploid male cells relative to their previous stage shown in Fig. 1c. H3K36me3 and DNA methylation data are not available in BM. Upregulated genes are shown in blue, and downregulated genes are shown in orange. Metaplots in this study were generated using deepTools software, with BPM (Bins Per Million mapped reads) as the normalization method. **a** Mi vs Me; **b** UM vs Mi; **c** BM vs UM; **d** SP vs BM; **e** Sp vs UM

In Mi relative to Me, the upregulated genes showed clear increases of broad H3K4me3 and ATAC signals but unchanged H3K27me3 or H3K36me3, these genes are not marked by gbM (Fig. 5a). By contrast, the downregulated genes in Mi that displayed gbM, showed no clear change of the histone marks (Fig. 5a). We hypothesized that the downregulation in Mi might be a consequence of the decay of Me transcripts rather than transcriptional repression [25, 26]. Interestingly, the upregulated genes in UM relative to Mi (or in BM relative to UM) showed little variation of the histone modifications (Fig. 5b, c), Similarly, genes upregulated in Sp showed little variation of histone modifications compared to UM or BM (Fig. 5d, e). This suggested that the Mi chromatin state was maintained for gene activation during male gamete development. By contrast, genes downregulated in Sp (relative to BM or UM) showed clear losses of H3K36me3, sharp H3K4me3 at TSS, and ATAC signals and were enriched for gbM (Fig. 5d, e). Thus, genes repressed in Sp displayed a distinct chromatin signature from those activated in Mi, UM, BM and Sp.

### Function of histone methyltransferase genes on Mi gene expression and function

**Fig. 6:**
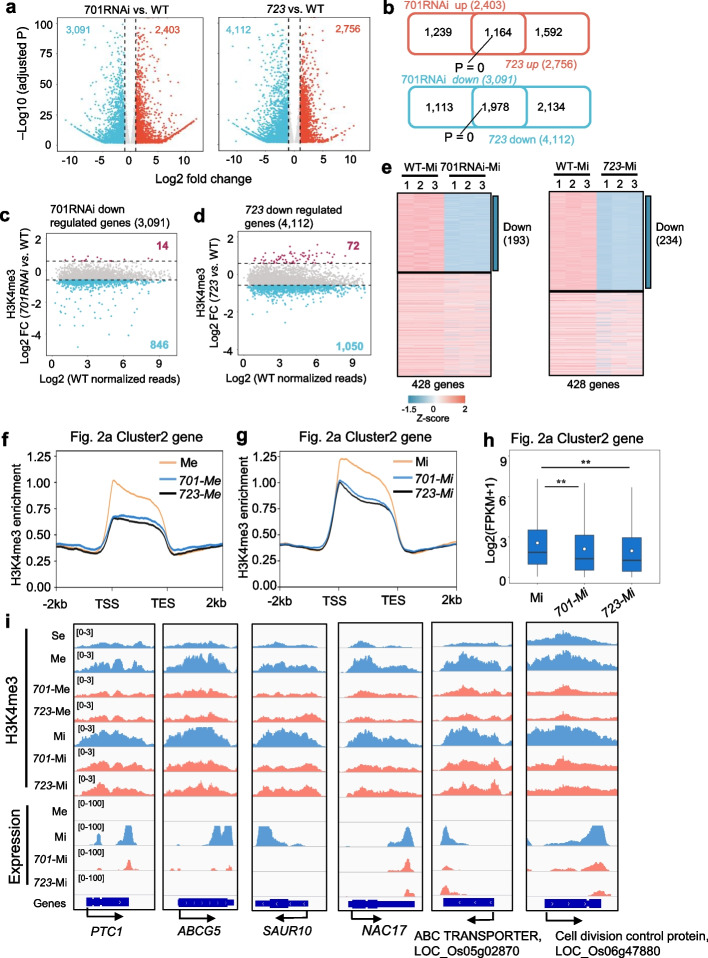
SDG701 and SDG723 are required for the pre-establishment of H3K4me3 in meiocyte and gene expression in Microspore. **a** Differentially expressed genes (DEGs) in SDG701-RNAi (701RNAi) and *sdg723* (*723*) mutant Mi cells relative to WT Mi cells; fold change (FC) > 2 and adjusted *p*-value < 0.01. **b** Overlaps of the DEGs in 701RNAi and *723* Mi cells. The *P*-values were calculated by hypergeometric tests. **c** and **d** Scattering plots of H3K4me3 changes in downregulated genes in 701RNAi (d) and *723* (e) Mi cells. The x axis represents H3K4me3 level (log2 normalized read counts use RPKM), and the y axis represents log2 fold change (FC mutant/wild type) of the mark. Hyper-H3K4me3 or hypo-H3K4me3 genes are marked by red and blue, respectively. **e** Expression levels of the 428 Mi-specific genes in 701RNAi and *723* mutant Mi cells. **f** and **g** Metaplots of H3K4me3 levels of cluster 2 genes (3,519, Fig. 2a) in 701RNAi (701) and *723* mutant Me (**f**) and Mi (**g**) cells compared to the wild type. **h** Boxplot showing the expression levels of cluster 2 genes (3,519, Fig. 2a) in 701RNAi (701) and *723* mutant Mi cells. Significance was calculated by t-test (** *P* < 0.01, two-tailed). **i** IGV screenshots of genes selected from **e**

To further study whether the H3K4me3 enhancement in Mi is important for Mi gene expression and function, we analyzed the transcriptome and genome-wide H3K4me3 in Mi and Me cells of mutant or RNAi lines of the H3K4me3 methyltransferase genes (*SDG701* and *SDG723*) [41, 42] (Additional file 2: Tables S1, S6). There were 3,091 to 4,112 downregulated and 2,403 to 2,756 unregulated genes in Mi cells of SDG701-RNAi and *sdg723* mutants (Fig. 6a), with half of the DEGs overlapping between the RNAi and the mutant cells (Fig. 6b). Approximately ⅓-¼ downregulated genes showed decreased H3K4me3 in the RNAi and the mutant (Fig. 6c, d). Importantly, about 50% of the Mi-specific genes were repressed in the RNAi or the mutant Mi cells (Fig. 6e). Thus, the SDG701 and SDG723-mediated H3K4me3 deposition in Mi promotes gene activation in the cell. To study whether *SDG701/723* genes were involved in the gain of H3K4me3 detected in the Me, we analyzed the H3K4me3 levels of cluster2 genes (from Fig. 2a) in the mutants. We found that these genes showed largely reduced H3K4me3 level in both Me and Mi cells of the mutants, and significantly decreased expression in the mutant Mi cells (Fig. 6f-i). The mutants also displayed defects in male gametogenesis and pollen development (Additional file 1: Fig. S12). These results indicate that the SDG701 and SDG723 methyltransferases are involved in pre-establishment and maintenance of H3K4me3 required for instructing subsequent gene expression during male gametogenesis.

## Discussion

**Fig. 7:**
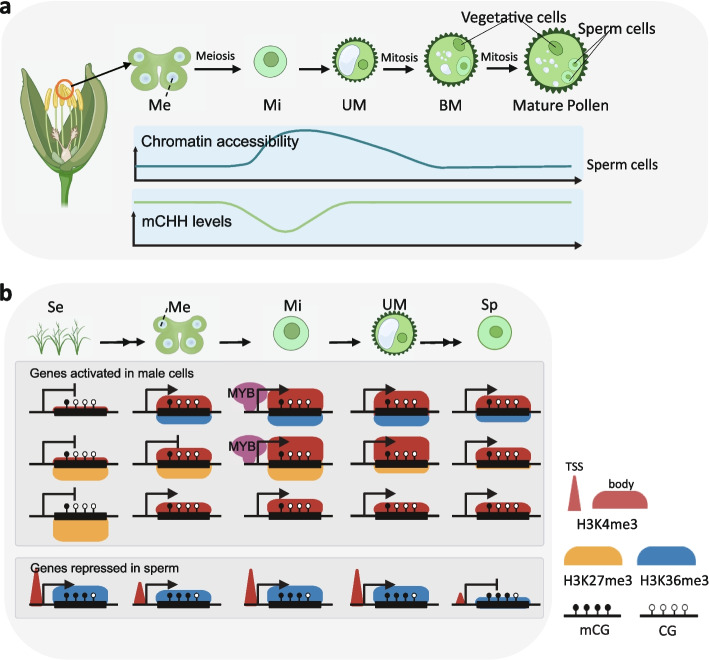
Chromatin regulation of male gamete gene expression during male gamete development. **a** Evolution of chromatin accessibility and mCHH levels during male gamete development. In undifferentiated microspore (Mi) that exhibits high enhanced open chromatin and reduced mCHH. **b** Pre-meiotic chromatin resetting for male gametic expression. Upper panels, histone methylation resetting at Me is required for gametic gene activation: with deposition of broad H3K4me3 and H3K36me3 in the body region, or with deposition of broad H3K4me3 in genes marked by H3K27me3 that is gradually reduced during male gamete development; or with removal of H3K27me3 and deposition of H3K4me3. Chromatin signature of haploid genome activation in microspore (Mi), consisting of globally enhanced chromatin accessibility and increased broad H3K4me3 and depletion of mCG from gene body. A possible role of MYB transcription factors (i.e. MYB80) in triggering gametic gene expression. Lower panel: genes repressed in sperm are decorated with sharp H3K4me3 at TSS, H3K36me3 and body mCG in Se and the early stages of male cell development but lose the activating histone methylation marks in sperm and become repressed

Our study shows that gene expression is continuously reconfigured during rice male germline development, producing functionally relevant and cell type-specific transcripts. However, H3K4me3, H3K27me3 and H3K36me3 profiles are already set up at prophase I of meiosis and maintained, or even enhanced in some cases, during microspore differentiation (Fig. 7b). Similarly, mCHH (located mainly in genic regions in the rice genome [43, 44]) is augmented in meiocyte and maintained during male germline development, except in undifferentiated microspores (Fig. 7a). The significant increase in mCHH in meiocyte may be partly due to methylation directed by siRNAs imported from tapetum, similar to what observed in Arabidopsis [11]. In contrast, the decrease of mCHH in early microspore is likely due to dilution by meiotic division and lagged maintenance by siRNA-directed DNA methylation in dividing cells [45], although active demethylation is not excluded. Thus, chromatin states of male germline gene expression are pre-established in meiocytes to poise genes for regulation during the gamete development. In addition, decreases of mCHH are detected in both up- and downregulated genes in the microspores. Thus, the remodeling of histone modifications appears to play a primary role in chromatin regulation of male cell gene expression. Our data showing that many genes with H3K4me3 variation and differential expression during microspore differentiation are also marked by H3K27me3 support the bivalent gene expression model observed in Arabidopsis male germ cells [20]. It is proposed that bivalent genes are in a poised state in pluripotent cells such as mammalian embryonic stem cells and in rice root meristem [46–48], allowing either activation or repression following differentiation cues. Possibly, bivalent genes in microspores may play a role in conferring the ability to regenerate haploid plants by in vitro culture. The mutation effects of *SDG701/723* genes on H3K4me3 in Me and Mi, Mi-specific gene expression, and microspore regeneration capacity (Additional file 1: Fig. S12b, c, d) support this hypothesis. In addition, the preferential expression of genes without gbM in microspores, which is also observed in stress-responsive bivalent genes in rice [49], may enhance the plasticity for regulation by cellular signals, as gbM mainly marks housekeeping and constitutively expressed genes [50]. Although the chromatin profiles already set up in meiocyte are maintained or enhanced in the haploid male cells, levels of the histone methylations and ATAC signals are generally reduced in sperm. Many genes decorated with sharp H3K4me3 at TSS and H3K36me3 in gene body as well as with gbM show decreases of the activating histone methylation marks and become repressed in sperm. H3K36me3 variation appears to play a primary role in the large shift of gene expression profile observed in Sp. This exacerbation of sperm chromatin remodeling, probably related to the nuclear compaction, may also play a role in epigenetic resetting and in instructing post-fertilization gene expression.

During male gamete development, many genes are transcribed after meiosis to ensure the completion of the entire haploid male gamete development [51, 52]. It is reported that in maize the haploid genome activation occurs before BM stage [26], while new transcripts could be detected in microspores at early stages [27]. The identification of stage-specific transcripts during microspore differentiation, which are enriched for specialized male cell functions, suggests that gene expression in male gamete cells is sustained to support male gametophyte development, although persisting sporophyte transcripts may also contribute to microspore differentiation. The decondensed chromatin state in Mi (with overall enhanced H3K4me3 and ATAC signals and low mCHH) might facilitate gametic gene expression. The enrichment of MYB binding sequences in microspore-specific ATAC sites and the cell-type specific expression of several MYB genes in the early microspores, together with previous data showing requirement of rice MYB80 for microspore development [40], suggest that MYB factors may play a critical role in triggering gametic gene expression in rice (Fig. 7b).

The observation that upregulated genes in the microspores and sperm show no gbM, while those repressed in male gametes, especially in sperm, are enriched for gbM suggests that genes without gbM are more exposed for selection during male gamete development (Fig. 7b). It is shown that genes with gbM are slowly evolving, with a lower nonsynonymous to synonymous substitution (*dN/dS*) ratio than those without gbM [53, 54]. This can be also observed in the rice genes (Additional file 1: Fig. S13). In addition, the up-regulated genes (without gbM) in Mi versus Me or in Sp versus UM displayed higher *dN/dS* ratios than the downregulated ones (with gbM) (Additional file 1: Fig. S13). Thus, selective upregulation of genes without gbM and downregulation of those with gbM in the male gametic cells might increase the selection time frame and scope at the whole haploid stage, which may contribute to reducing inbreeding depression [55, 56], and to increasing offspring fitness [57, 58].

## Conclusions

In summary, our analysis of cell-type specific transcriptomes and epigenomes across rice male germline developmental process reveals that chromatin state in meiocytes is stably maintained, and enhanced in some cases, during male gametogenesis to instruct male cell-type specific gene expression. The meiocyte chromatin state is characterized by increases of H3K4me3, decreases of H3K27me3 and increases of mCHH. This chromatin profile is maintained during male gametogenesis, except in early microspore where H3K4me3 is further enhanced while mCHH, as well as mCHG, is reduced, which may be involved in initiating gametic gene expression after meiosis. Notably, male gametic gene expression preferentially activates non-constitutive genes without gbM, which may have implication in haploid/pollen selection and may reduce inbreeding depression while enhancing offspring fitness.

## Methods

### Plant materials and growth conditions

Rice varieties used in this study are Zhonghua11 (ZH11, *Oryza sativa* spp.* japonica*)*.* The mutant materials of *jmj705* were obtained from a previously published study [59]. The SDG701-RNAi, plants were described previously [41]. *SDG723* CRISPR/Cas9 mutant plants were produced in the ZH11 background. The selected sgRNA target was GGTGAACTCGAACCATTGAA. For field cultivation, the plants were grown in Wuhan during summer seasons under standard agricultural practices. Seedlings were transplanted to the field in early May and harvested in October. For greenhouse experiments, germinated rice seedlings were planted in soil-filled containers and grown under controlled conditions with a 14-h light/10-h dark cycle at 32 °C (light) and 26 °C (dark). Tillers of rice plants at an appropriate developmental stage were harvested for collection of spikelets. The collected spikelets were used for phenotypic analysis and isolation of male germ cells.

### Isolation of male germ cells

For collection of ZH11 spikelets with meiocytes at prophase of meiosis I, tillers whose flag leaf auricle was about 5 cm under the auricle of the penultimate leaf were chosen and 0.2–0.3 cm long fresh spikelets were collected. A spikelet usually develops only one glumous flower, a glumous flower contains 6 anthers, and the developmental stages of each anther are consistent. Meiocytes at early prophase I were collected as previously described by Jiang et al. [60]. In brief, gentle pressure was applied to the anthers to allow the separation of wormlike cell clusters from anther walls under the inverted microscope. Then, meiocytes were collected with capillary glass pipettes without taking any somatic cells.

For collection of tetrads and microspores, when tillers flag leaf collar and penultimate leaf collar aligned spikelets of 0.4–0.6 cm length were taken. Like extracting meiocytes, anthers were dissected out and gentle pressure was applied to allow the release of germ cells. Cells with tetrad shapes were collected as tetrads. Cells with single, free-floating state and have not formed cell walls were collected as microspores. Isolating intact tetrads in rice is technically challenging, as during harvest/sampling, the tetrad cells usually detach from each other (indistinguishable from early micropores), making it difficult to obtain sufficient amounts of intact tetrads for histone epigenome profiling (> 10,000 cells are required for CUT&Tag), so we had chosen only to use the early microspore stage for epigenomic profiling.

For collection of mature unicellular microspores, when tillers flag leaf auricle was about 5 cm above the auricle of the penultimate leaf, fresh spikelets > 0.6 cm were collected. At this stage the color of the spikelets is still white. Anthers were dissected out and gentle pressure was applied to allow the release of germ cells. Cells with distinct germination pores and having formed cell walls are collected as unicellular microspores.

For collection of bicellular microspores, spikelets turning greener in color were taken. Anthers were dissected out and gentle pressure was applied to allow the release of germ cells. Cells with distinct large vacuoles, two nuclei, and thickened pollen walls are collected as bicellular microspores.

For collection of mature pollen, tillers green spikelets were taken. At this point, the anthers have developed into yellow color. Anthers were dissected out and gentle pressure was applied to allow the release of germ cells. The cells that are distinctly filled with starch are collected as pollen.

### Isolation of sperm cells

A differential centrifugation isolation method was used to separate sperm from whole rice anthers with minor modifications [24]. In detail, approximately 400 mature anthers were soaked in 3 mL of 0.33 M mannitol for 30 min and then transferred into 3 mL chilled 12% (w/v) sucrose to release sperm at 4 °C for 1 h. The suspensions were sequentially filtered through 20 µm and 10 µm Nylon Membrane Filters (Millipore, Cat. # NY2004700, Cat. # NY1004700), followed by addition of an equal volume of chilled 60% Percoll (containing 18% w/v sucrose) with gentle mixing. The sperm cell suspension was sequentially loaded with 3 mL each of chilled 20% Percoll (containing 15% w/v sucrose) and chilled 5% Percoll (containing 15% w/v sucrose), followed by centrifugation at 3,000 g at 4 °C for 45 min. The sperm cell-rich fraction will form a band at the 5/20% interface. The sperm cell enriched fraction was mixed with an equal volume of chilled 15% sucrose solution and centrifuge at 5,000 g at 4 °C for 10 min. The sperm cell pellet was collected from the bottom 100 μL of the mixed solution. Isolated sperm were checked by 4′, 6-diamidino-2-phenylindole dihydrochloride (DAPI) staining.

### Isolation of seedling cell nuclei

To preserve the integrity of tissue nuclei, we employed the "knife cutting method" for mass extraction of intact nuclei from seedling. Fourteen-day-old rice tissues (0.5 g, green part) were chopped until homogenized using scissors or a blade and then suspended in 10 mL of 1 × PBS. The homogenate was filtered through Miracloth (Millipore, cat. # 3,536,029). The extract was centrifuged at 600 g at 4 °C for 10 min. The resulting pellet was lysed with M2 buffer (1 × PBS, 0.1 M NaCl, 1 M hexylene glycol, 10 mM MgCl_2_, 0.5% Triton X-100, 10 mM β-mercaptoethanol, 1 × protease inhibitor), and the nuclei pellet was resuspended in 1 × PBS for library construction.

### Cytological analysis

The experiments were performed as described by Liu et al. [61]. Briefly, young panicles of various lengths were fixed in Carnoy’s solution (ethanol:glacial acetic acid, 3:1 [v/v]). Then, germ cells were stained with DAPI to observe different developmental stages. Slides were examined on an Olympus BX61 epifluorescence microscope and imaged with a CCD Olympus DP71 camera. Adobe Photoshop CS5 software was applied for image processing.

### RNA-seq and BS-seq library construction

Isolation and purification of mRNA from different types of cells were performed using the Single Cell Full Length mRNA Amplification Kit (Vazyme, Cat. # N712). The RNA-seq libraries were prepared using the TruePrep DNA Library Prep Kit V2 for Illumina (Vazyme, Cat. # TD502) and sequenced on Illumina HiSeq 6000. For rice in SY63 backgrounds, we used single male germ cell to construct the transcriptome library. We used approximately 100–200 male germ cells to construct the transcriptome library.

BS-seq libraries were constructed using reported protocol Clark et al. [62]. About 400–500 male germ cells were pooled for each replicate bisulfite seq library construction.

### Antibodies

The antibodies were as follows: anti-H3K4me3 (Abcam, ab8580), anti-H3K9me2 (Abcam, ab1220), anti-H3K27me3 (Diagenode, C15410195), anti-H3K36me3 (Active Motif, 61,021), anti-H3 (Abcam, ab1791).

### CUT&Tag library construction

The CUT&Tag libraries were prepared using the Hyperactive Universal CUT&Tag Assay Kit for Illumina (Vazyme, Cat. # TD903) and sequenced on Illumina HiSeq 6000. We used approximately 10,000 male germ cells to construct the CUT&Tag library by male germ cell isolation technology, followed by centrifugation at 600 g at 4 °C for 5 min. The male germ cell pellets were gently resuspended in 200 µL of ice-cold Nuclear Extraction Buffer 2 (NEB2) (0.25 M sucrose, 10 mM Tris–HCl pH 8.0, 10 mM MgCl_2_, 1% Triton X-100, 1 × protease inhibitor), and incubated on ice for 15 min. The nuclei suspension followed by centrifugation at 600 g at 4 °C for 5 min. The nuclei pellet washed with wash buffer (20 mM HEPES, pH 7.5, 150 mM NaCl, 0.5 mM spermidine) and immobilized to concanavalin A-coated beads (Vazyme, TD903) with incubation at room temperature for 20 min. The bead-bound nuclei were incubated in 200 µL of primary antibody buffer (wash buffer with 1% BSA, 2 mM EDTA and 0.05% digitonin for gentle permeabilization of the nuclear membrane) with a 1:50 dilution of anti-H3K4me3 antibody (Abcam, ab8580) or other antibodies at 4 °C by rotating overnight. The next day, the primary antibody buffer was removed, and the cells were washed with 800 µL of dig-wash buffer (wash buffer with 1% BSA and 0.05% digitonin) three times. After washing, the antibody-incubated nuclei were resuspended in 200 µL of dig-wash buffer with a 1:100 dilution of secondary antibody (Vazyme, TD903) and incubated at room temperature with gentle rotation for 1 h. To eliminate binding antibodies, the nuclei were washed three times with 800µL of dig-wash buffer. After a brief wash with dig-wash buffer, the nuclei were resuspended in 200 µL of dig-300 buffer (20 mM HEPES, pH 7.5, 300 mM NaCl, 0.5 mM spermidine, 1% BSA and 0.01% digitonin) and incubated at room temperature for 1 h with gentle rotation. pA-Tn5-bound nuclei were washed with 800 µL of dig-300 buffer three times and then tagmentated at 37 °C for 1 h. 15 mM EDTA, 500 µg/ml proteinase K and 0.1% SDS were added after tagmentation to terminate tagmentation and degrade protein. Genomic DNA was extracted and purified with VAHTS DNA Clean Beads (Vazyme, N411-01). Purified genomic DNA was amplified using the universal i5 primer and barcoded i7 primer (Vazyme, TD202) using Vazyme Master Mix (Vazyme, TD903). The library PCR products were cleaned with Agencourt AMPure XP beads (Beckman Coulter, A63881) and sequenced on Illumina HiSeq 6000. we isolated approximately > 10,000 male germ cells to construct the CUT&Tag library. About 100,000 sperm cells were pooled for each replicate CUT&Tag library construction. About 100,000–200,000 seedling cell nuclei were pooled for each replicate CUT&Tag library construction.

### ATAC-seq library construction

For ATAC-seq library construction, the Hyperactive ATAC-Seq Library Prep Kit for Illumina (Vazyme, Cat. # TD711) was used to prepare libraries for different types of cells. Using the same number of cells as for CUT&Tag, we isolated approximately 10,000 male germ cells to construct the ATAC-seq library. Approximately 100,000 sperm cells and 100,000–200,000 seedling cell nuclei were pooled for each replicate ATAC-seq library construction.

### Identify specifically expressed genes

Stage-specific genes based on expression data were detected by calculating the specificity measure (SPM), Using the methods described in the references [63]. For each gene, we calculated the average FPKM value in each sample. Then, the SPM value of a gene in a sample was computed by dividing the average FPKM in the sample by the sum of the average FPKM values of all samples. The SPM value ranges from 0 (a gene is not expressed in a sample) to 1 (a gene is fully sample-specific).

### RNA-seq data analysis

RNA-seq raw reads were filtered by fastp (v.0.23.4) [64] to remove low quality reads and adapter. Clean reads were aligned to the MSU7.0 rice reference genome (Rice Genome Annotation Project, http://rice.plantbiology.msu.edu/) by hisat2 (v.2.1.0) [65]. FeatureCounts (v.2.0.1) [66] were used for quantitative analysis and the differentially expressed genes were calculated by DESeq2 [67]. To identify stage-specific gene expression changes between adjacent developmental stages, both up- and down-regulated genes were analyzed. Specifically, we used the following filtering parameters: |fold change|> 2, adjusted *p*-value < 0.01, with FPKM > 3 for upregulated genes and FPKM < 1 for downregulated genes. Gene Ontology (GO) enrichment analysis was performed using TBtools-II [68]. Significantly enriched GO terms were identified based on the hypergeometric test with a significance threshold of *p*-value < 0.01. Only GO terms containing at least 3 mapped genes were considered in the analysis.

### CUT&Tag data analysis

Fastp (v.0.23.4) was used to remove low-quality reads and adapter from the CUT&Tag raw data. Clean reads were mapped to the MSU7.0 genome by Bowtie2 (v.2.4.1) [69]. SAMtools (v.1.20) [70] is used to convert between SAM and BAM formats, sort and deduplicate BAM files, and merge two biological replicates. MACS2 (v.2.2.7.1) [71] was used to call histone modification peaks with the parameters (-f BAMPE -g 3.8e8 –broad -q 0.01 for H3K27me3, H3K36me3 and H3K9me2; -f BAMPE -g 3.8e8 -q 0.01 for H3K4me3), and H3 sample was used as control. The bigwig files were generated using bamCoverage with the parameters (-bam -binSize 10 –normalize Using BPM) in deepTools (v.2.5.3) [72], which were visualized in the Integrated Genome Browser, draw heatmaps and metaplots. Histone modification regions were defined based on the merged peak regions from replicate samples across different cell types. Differential analysis of histone signals was performed using the DESeq2 package in R, with the criteria of an adjusted *p*-value < 0.01 and a fold-change > 1.5 to identify significantly differential histone modifications.

### BS-seq data analysis

BS-seq low-quality reads were filtered out from the raw data by TrimGalore (v.0.6.6; http://www.bioinformatics.babraham.ac.uk/projects/trim_galore/). Clean reads were mapped to the MSU7.0 rice genome. Bismark (v.0.23.1) [73] was used to match, deduplicate and extract methylation sites. To obtain more loci for analysis, we combined the two biological replicates. Duplicates were removed and uniquely mapped reads were retained and each cytosine covered by at least five reads for further analysis. Density plots show the frequency distribution of DNA methylation differences between 50-bp window of two samples with at least 20 informative sequenced cytosines in both samples and 70% CG, 30% CHG, or 10% CHH methylation in either of the samples as previously described [74].

To identify differential methylated regions (DMRs), the whole genome was divided into 100-bp bins. Bins that contained at least five cytosines each with every cytosine with at least a five-fold coverage were retained, absolute methylation difference of 0.6, 0.3, and 0.1 for CG, CHG, and CHH, respectively, and *P* values < 0.01 (Fisher’s exact test) were considered as DMRs.

### ATAC–seq data analysis

Fastp was used to remove low-quality reads and adapters from the ATAC raw data. Clean reads were mapped to the MSU7.0 genome by Bowtie2 (v.2.4.1). SAMtools (v.1.20) is used to convert between SAM and BAM formats, sort and deduplicate BAM files, and merge two biological replicates. MACS2 (v.2.2.7.1) was used to call histone modification peaks with the parameters (-f BAMPE -g 3.8e8 –nomodel –shift −100 –extsize 200 -q 0.01). The bigwig files were generated using bamCoverage with the parameters (-bam -binSize 10 –normalize Using BPM) in deepTools (v.2.5.3). To identify transcription factor binding motifs significantly enriched in ATAC-seq peaks, we performed de novo motif discovery and known motif enrichment analysis using HOMER (v4.11.1) [75].

## Supplementary Information

Additional file 1: Figure S1. Flowchart of germ cell sampling rice male gamete development and library construction. Figure S2. Analysis of rice male cell RNA-seq data. Figure S3. Transcript levels of meiosis marker genes during early male gamete development. Figure S4. Analysis of histone methylation CUT&Tag data of rice Se, Me and haploid male cells. Figure S5. DNA methylation dynamics during male gamete development. Figure S6. Analysis of transposable elementexpression during male gamete development. Figure S7. Detailed analysis of genes with H3K4me3 variation during male germ line development. Figure S8. H3K27me3 dynamics during male gamete development. Figure S9. H3K36me3 dynamics during male gamete development. Figure S10. H3K9me2 is relatively stable during male gamete development. Figure S11. Analysis of genes that gained chromatin accessibility in Me and haploid male cells. Figure S12. Effects of H3K4me3 methyltransferase gene mutations on microspore development and callus regeneration in *in vitro* culture. Figure S13. Calculation of *d*_n_/d_s_ ratios of rice genes with and without gbM.

Additional file 2: Table S1. Rice single male cell type RNA-seq data. Table S2. Rice male cell type histone methylation CUT&Tag data. Table S3. Rice male cell ATAC-seq data. Table S4 Rice male cell BS-seq data. Table S5. TE expressionsof different categories in Se, Me, and haploid male cells. Table S6. H3K4me3 methyltransferase gene mutant microspore CUT&Tag data.

## Data Availability

All high throughput data in support of the findings of this study are deposited to the Gene Expression Omnibus (GEO) under the accession number GSE294738 [76]. The rice seedling BS-seq data are under the accession number GSE235680 [8]. The rice seedling ATAC-seq data are under the accession number GSE144564 [77]. The rice seedling ChIP-seq data are under the accession number GSE142570 [78].
