# Supplementary material for: Premeiotic chromatin states orchestrate gene expression during male gametogenesis in rice
Source: Genome Biol. 2026 Jun 12;27:192. doi: 10.1186/s13059-026-04129-4 (PMC13262433; doi:10.1186/s13059-026-04129-4)
Supplement: Supplementary file 1 — Additional file 1: Figure S1. Flowchart of germ cell sampling rice male gamete development and library construction. Figure S2. Analysis of rice male cell RNA-seq data. Figure S3. Transcript levels of meiosis marker genes during early male gamete development. Figure S4. Analysis of histone methylation CUT&Tag data of rice Se, Me and haploid male cells. Figure S5. DNA methylation dynamics during male gamete development. Figure S6. Analysis of transposable elementexpression during male gamete development. Figure S7. Detailed analysis of genes with H3K4me3 variation during male germ line development. Figure S8. H3K27me3 dynamics during male gamete development. Figure S9. H3K36me3 dynamics during male gamete development. Figure S10. H3K9me2 is relatively stable during male gamete development. Figure S11. Analysis of genes that gained chromatin accessibility in Me and haploid male cells. Figure S12. Effects of H3K4me3 methyltransferase gene mutations on microspore development and callus regeneration in in vitro culture. Figure S13. Calculation of dn/ds ratios of rice genes with and without gbM. [file 13059_2026_4129_MOESM1_ESM.pdf]

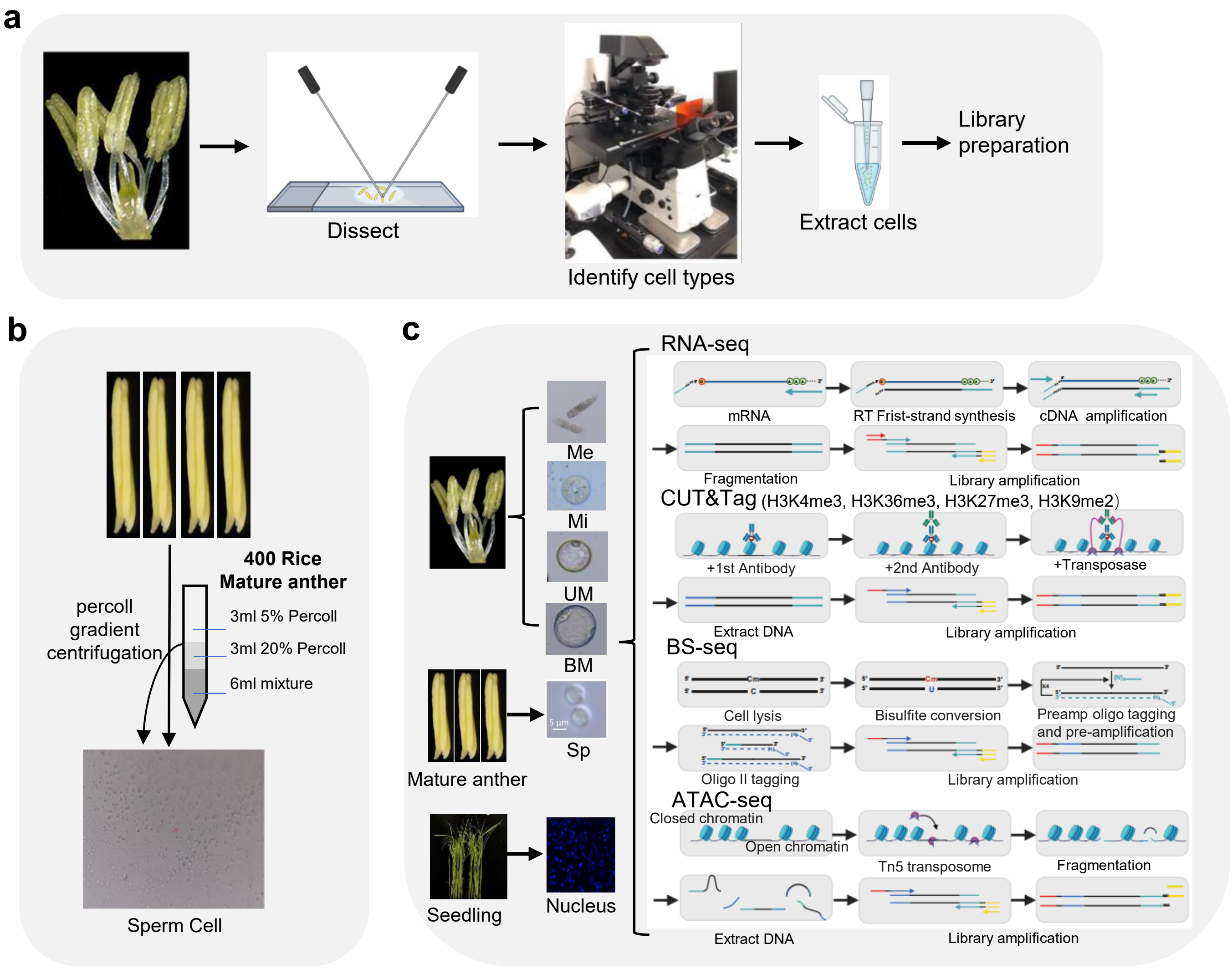

Fig S1. Flowchart of germ cell sampling rice male gamete development and library construction.

a, A schematic illustration of the operational workflow for sampling male germ cells under microscope. **b**, A flowchart for isolation and extraction of rice sperm cells (see method). **c**, Flowcharts of male cell high throughput sequencing library constructions for RNA seq, CUT&Tag, BS seq, and ATAC seq.

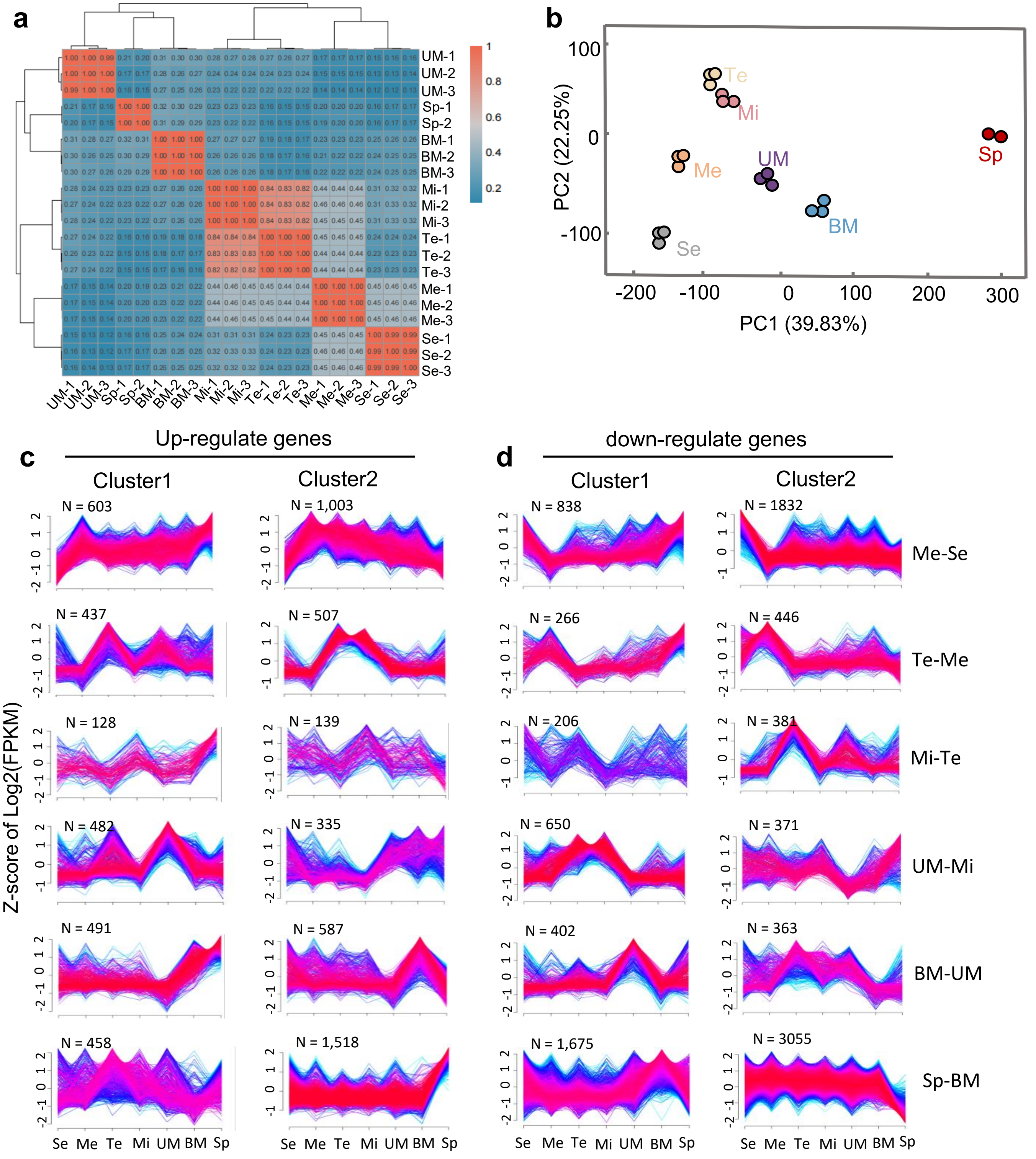

Fig S2. Analysis of rice male cell RNA-seq data.

a, Person correlation analysis of RNA seq reads from 3 replicates of seedling (Se), meiocytes (Me), tetrad (Te), and the microspores at different sages, and two replicates of sperm. **b**, Principal component analysis (PCA) of Se, Me and the haploid male cells. **c** and **d**, Relative expression levels in Se, Me and the haploid male cells of the up- and down-regulated genes in Me versus Se and the haploid cells versus their previous stage (shown in Fig. 1c). Each group is separated into two clusters based on their expression trend.

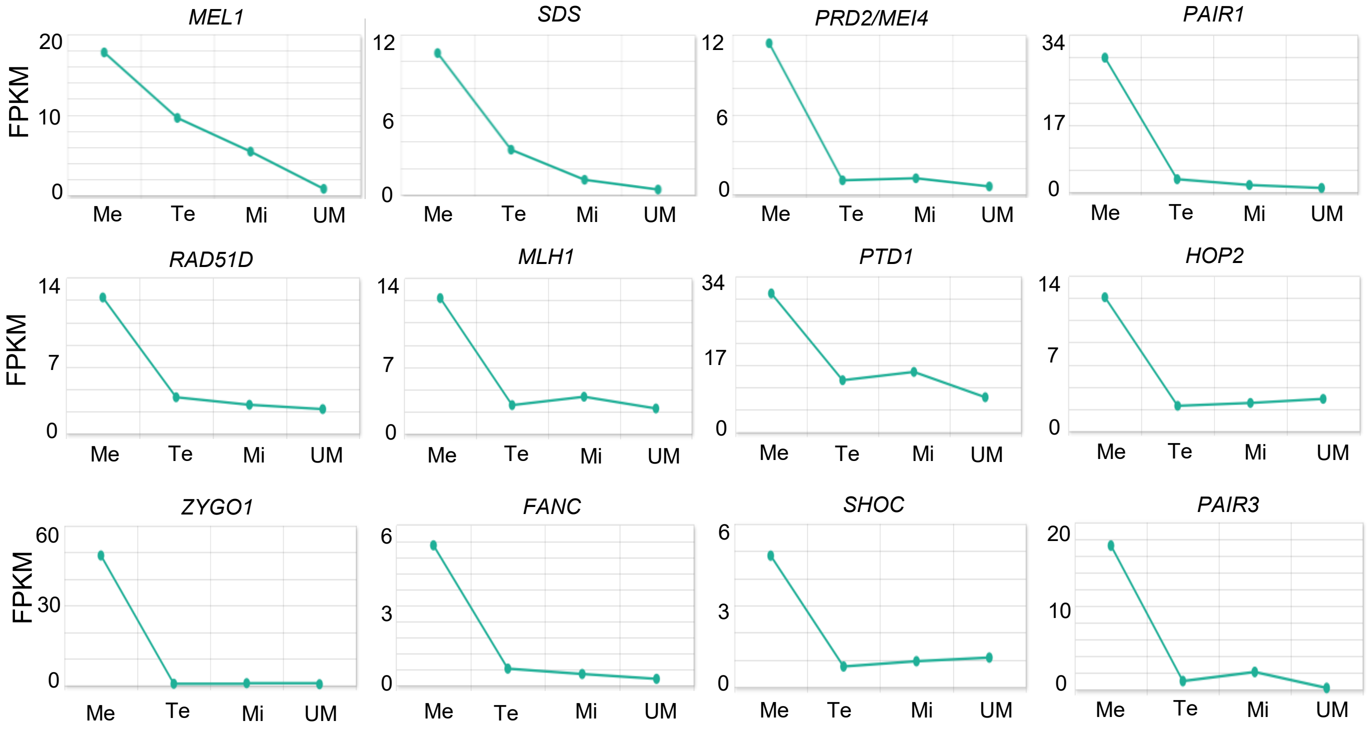

Fig S3. Transcript levels of meiosis marker genes during early male gamete development.

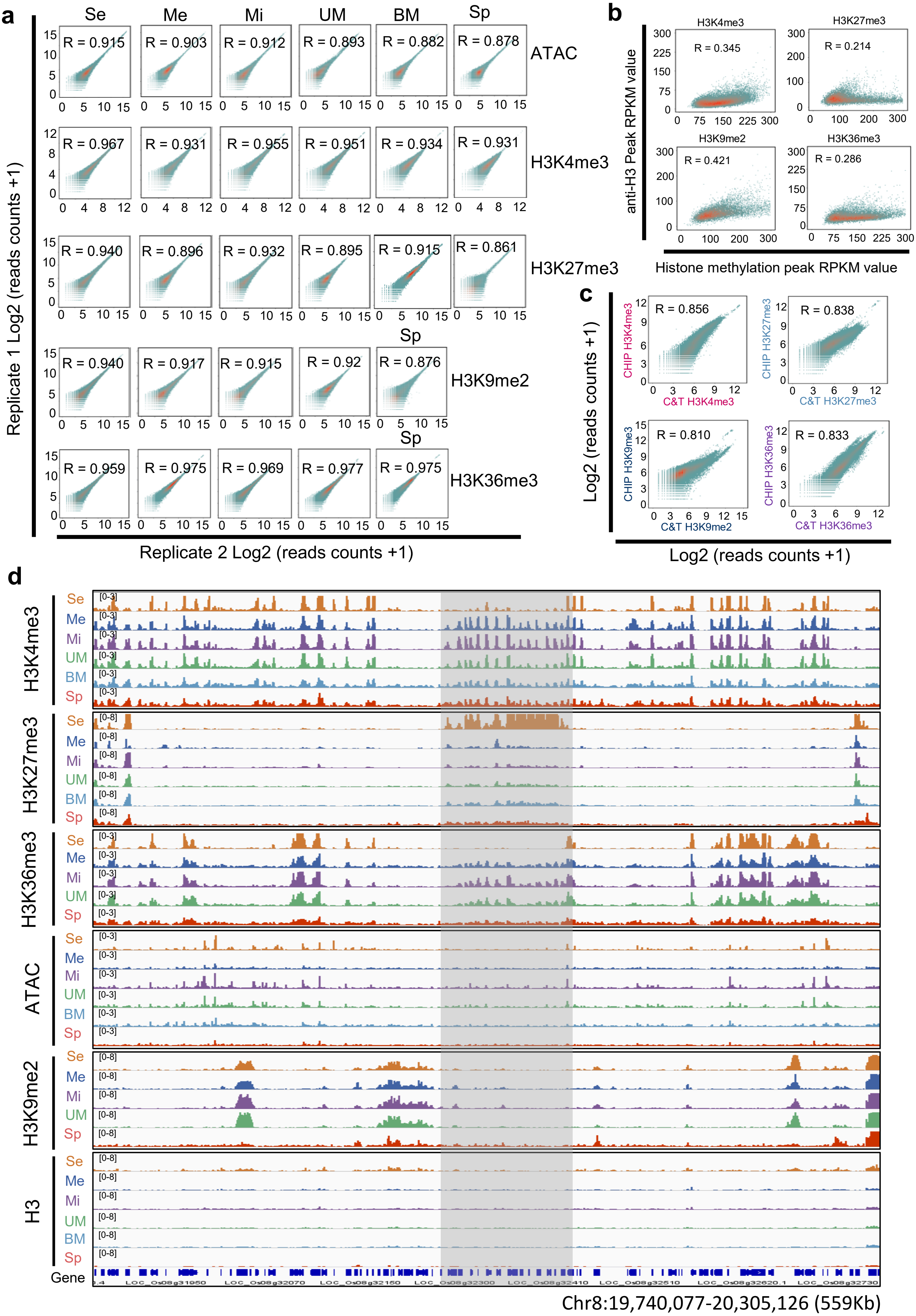
a
Se
Me
Mi
UM
BM
Sp
ATAC
H3K4me3
H3K27me3
H3K9me2
H3K36me3
R = 0.915
R = 0.903
R = 0.912
R = 0.893
R = 0.882
R = 0.878
R = 0.967
R = 0.931
R = 0.955
R = 0.951
R = 0.934
R = 0.931
R = 0.940
R = 0.896
R = 0.932
R = 0.895
R = 0.915
R = 0.861
R = 0.940
R = 0.917
R = 0.915
R = 0.92
R = 0.876
R = 0.959
R = 0.975
R = 0.969
R = 0.977
R = 0.975
Replicate 1 Log2 (reads counts +1)
Replicate 2 Log2 (reads counts +1)
b
H3K4me3
R = 0.345
H3K27me3
R = 0.214
H3K9me2
R = 0.421
H3K36me3
R = 0.286
anti-H3 Peak RPKM value
Histone methylation peak RPKM value
c
R = 0.856
R = 0.838
R = 0.810
R = 0.833
CHIP H3K4me3
C&T H3K4me3
CHIP H3K27me3
C&T H3K27me3
CHIP H3K9me3
C&T H3K9me2
CHIP H3K36me3
C&T H3K36me3
Log2 (reads counts +1)
Log2 (reads counts +1)
d
H3K4me3
H3K27me3
H3K36me3
ATAC
H3K9me2
H3
Gene
Chr8:19,740,077-20,305,126 (559Kb)

Fig S4. Analysis of histone methylation CUT&Tag data of rice Se, Me and haploid male cells.

a, Pearson correlation coefficients between biological replicates of CUT&Tag data from seedling (Se), meiocyte (Me) and the haploid male cells. Numbers of the mapped reads from each genomic bin (1 kb) were plotted in log scale between two replicates. **b**, Correlation analysis between histone methylation and the control (H3) CUT&Tag data obtained from Mi cells. **c**, Correlation analysis of CUT&Tag and ChIP-seq data of H3K4me3, H3K9me2, H3K27me3 and H3K36me3 obtained from seedling cells or tissues. Numbers of the mapped reads from each genomic bin (1 kb) were plotted in log scale between two replicates. **d**, Integrative Genomics Viewer (IGV) genome browser screenshots of H3K4me3, H3K27me3, H3K36me3, H3K9me2, and H3 of a genomic region in chromosome 8 in Se, Me and the haploid male cells. A region with clear changes of chromatin states from Se to Me and in the haploid male cells is shaded.

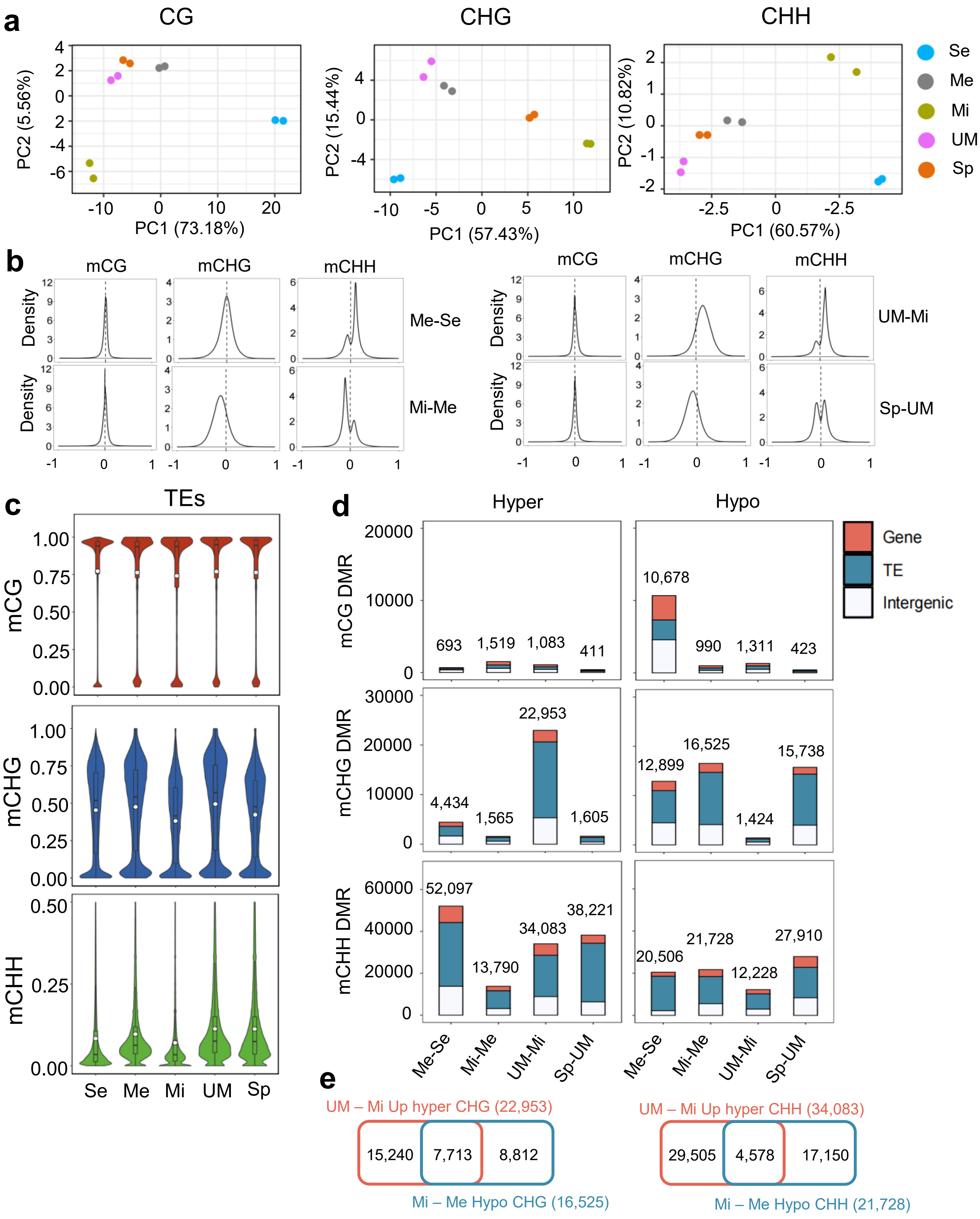

Fig S5. DNA methylation dynamics during male gamete development.

a, PCA analysis of DNA methylomes of Se, Me and haploid male cells, denoted by different colors. **b**, Density plots of frequency distribution of fractional methylation difference between the indicated comparisons. **c**, Violin plots showing overall cytosine methylation levels (mCG, mCHG, and mCHH) in transposable elements (TE) of Se, Me and haploid male cells. Values of the methylomes are averages from the two replicates. The average methylation levels (white dots) and median values (black bars) are indicated. **d**, Numbers of differentially methylated regions (DMRs) between the indicated comparisons, distributed in protein coding gene (red), TE (blue), and intergenic regions (white). **e,** Venn diagrams showing the overlap of hypo (decreased) DMRs of mCHG and mCHH in Mi versus Me and the hyper (increased) DMRs in UM versus Mi.

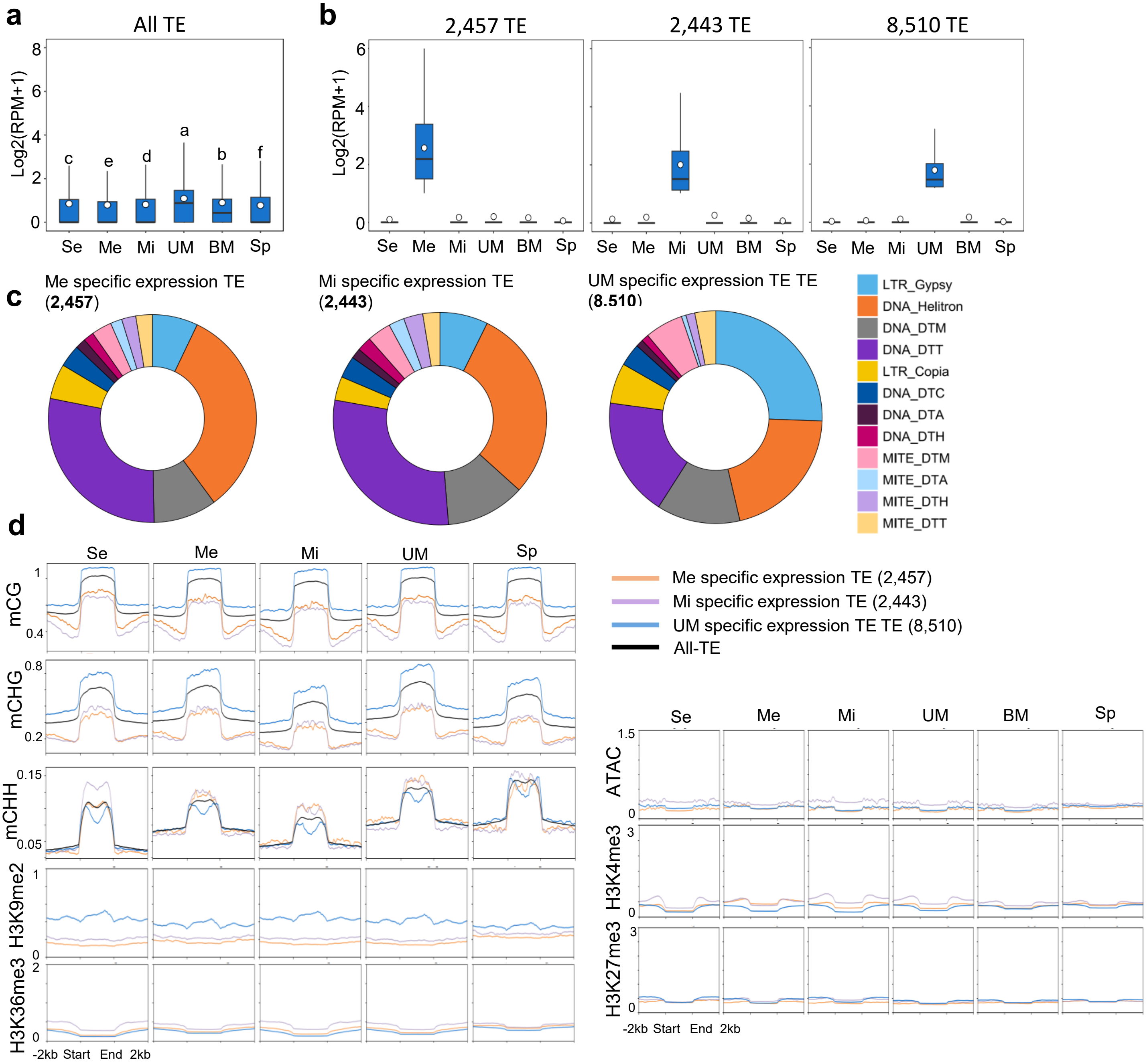

Fig S6. Analysis of transposable element (TE) expression during male gamete development.

a, Boxplot showing the expression levels of all TEs, excluding those not expressed in any stage. The significance was calculated with multiple comparison tests. Different letters on top of the bars indicate a significant difference ($p<0.01$). **b**, Boxplot showing the expression levels of specifically expressed (RPM > 1, SPM > 0.8) TEs in Me (2,457 TE), Mi (2,443 TE), and UM (8,510 TE). **c**, Pie chart showing the categories and proportions of expressed TEs in Me, Mi, and UM. **d**, Metaplots showing the DNA methylation, ATAC, and histone modification levels of expressed TEs in Me, Mi and UM (from figure c) in Se, Me, and haploid male cells. Averages of all TEs are shown as controls (blacklines).

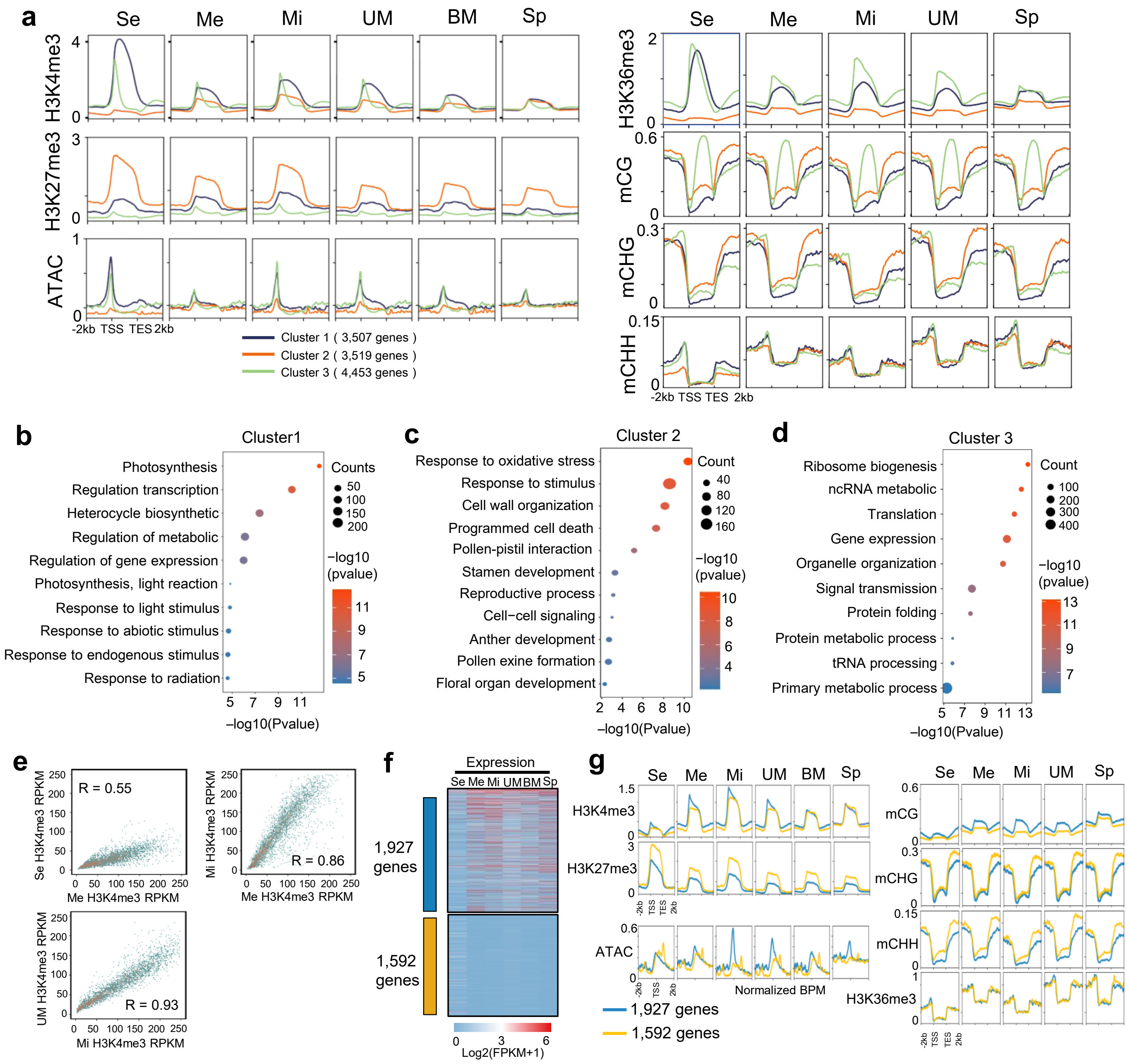

Fig S7. Detailed analysis of genes with H3K4me3 variation during male germ line development.

a, Metaplots of gene H3K4me3 levels in the indicated cell types. The clusters 1–3 genes are from Fig. 2a. H3K27me3, H3K36me3, ATAC, and DNA methylation (at CG, CHG, and CHH sites) levels of the H3K4me3-marked genes are shown. Metaplots in this study were generated using deepTools software, with BPM (Bins Per Million mapped reads) as the normalization method**. b-d,** GO enrichment analysis of clusters 1, 2, and 3 genes in Figure 2a. **e**, Correlation analysis of H3K4me3 of cluster 2 genes (Cluster 2 in Fig. 2a) in Me versus Se, Mi versus Me, and UM versus Mi. **f**, Clustering by expression level of the genes that gained H3K4me3 in Me and the haploid male cells. Among the 3,519 genes that gained H3K4me3 (Cluster 2 in Fig. 2a), 1,927 genes were expressed in Me and the haploid male cells, while 1,592 genes showed low or undetectable expression in Me and the haploid male cells (FPKM < 1). **g,** The metaplot shows the levels of H3K4me3, H3K27me3, H3K36me3, ATAC, and DNA methylation for 1,927 (blue) and 1,592 (orange) genes, respectively. The silent genes displayed higher H3K27me3 than the expression ones.

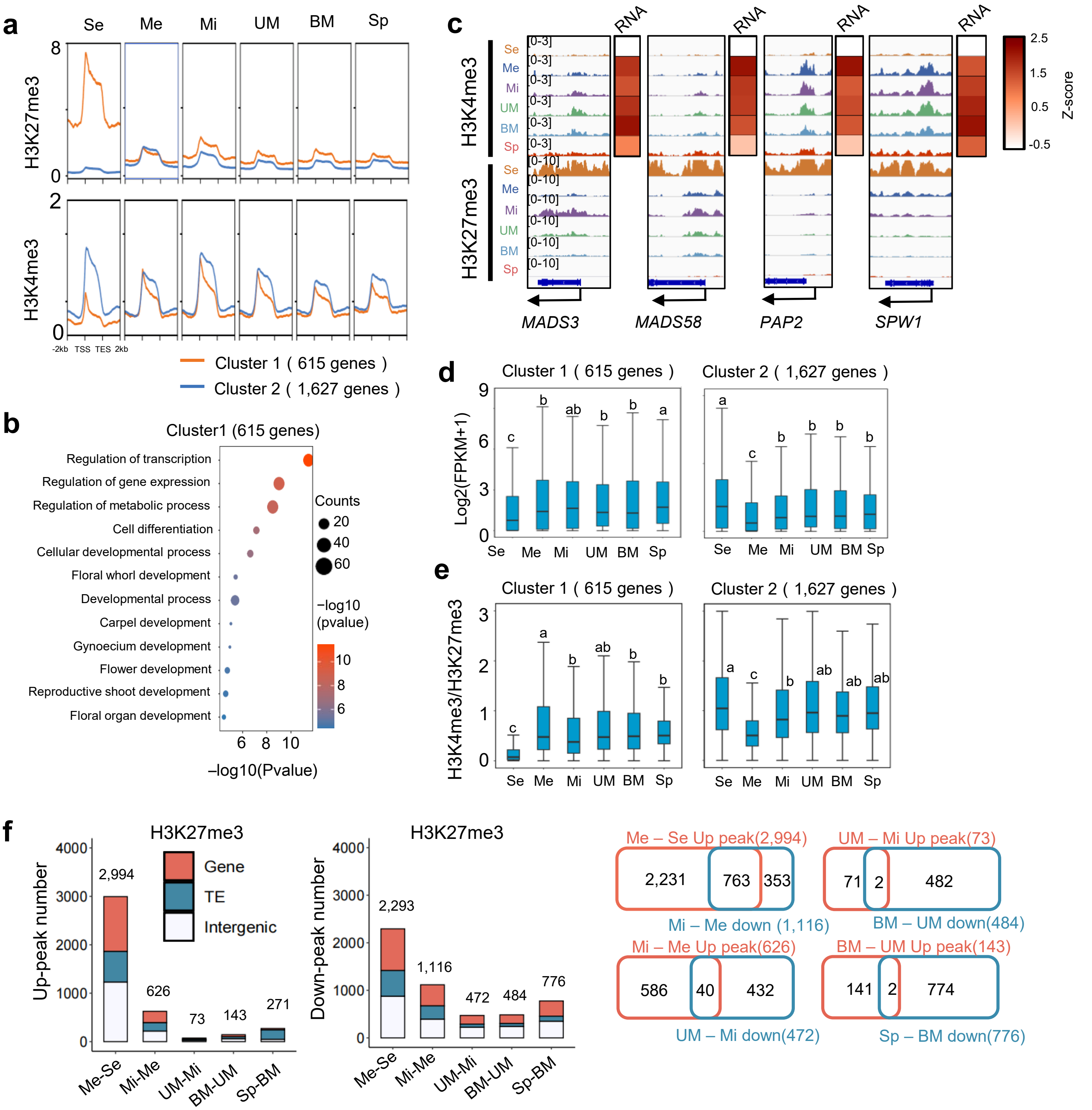

Fig S8. H3K27me3 dynamics during male gamete development.

a, Clustering by metaplots of genes with H3K27me3 changes during male gamete development. Cluster 1 (**orange**): H3K27me3 decreased starting from the Meiocyte (Me); Cluster 2 (blue): H3K27me3 increased in Me. The levels of H3K4me3 of the two cluster in the corresponding cell types are shown. **b**, Gene ontology term enrichment of Clusters 1 genes. **c,** Integrative Genomics Viewer (IGV) for marker genes selected from Clusters 1. **d**, The boxplot of expression levels of cluster 1 and 2 genes in the different cell types. The significance was calculated with multiple comparison tests. Different letters on top of the bars indicate a significant difference ($p<0.01$). **e,** The boxplot of H3K4me3/H3K27me3 ratios of the clusters 1 and 2 genes. The significance was calculated with multiple comparison tests. Different letters on top of the bars indicate a significant difference ($p<0.01$). **f**, Numbers of differentially methylation peaks between adjacent developmental stages (fold change > 1.5 ; adjusted p-value < 0.01) and overlaps between the downregulated peaks in the male cells (relative to the previous stage) with the upregulated ones in the previous stage or cell type.

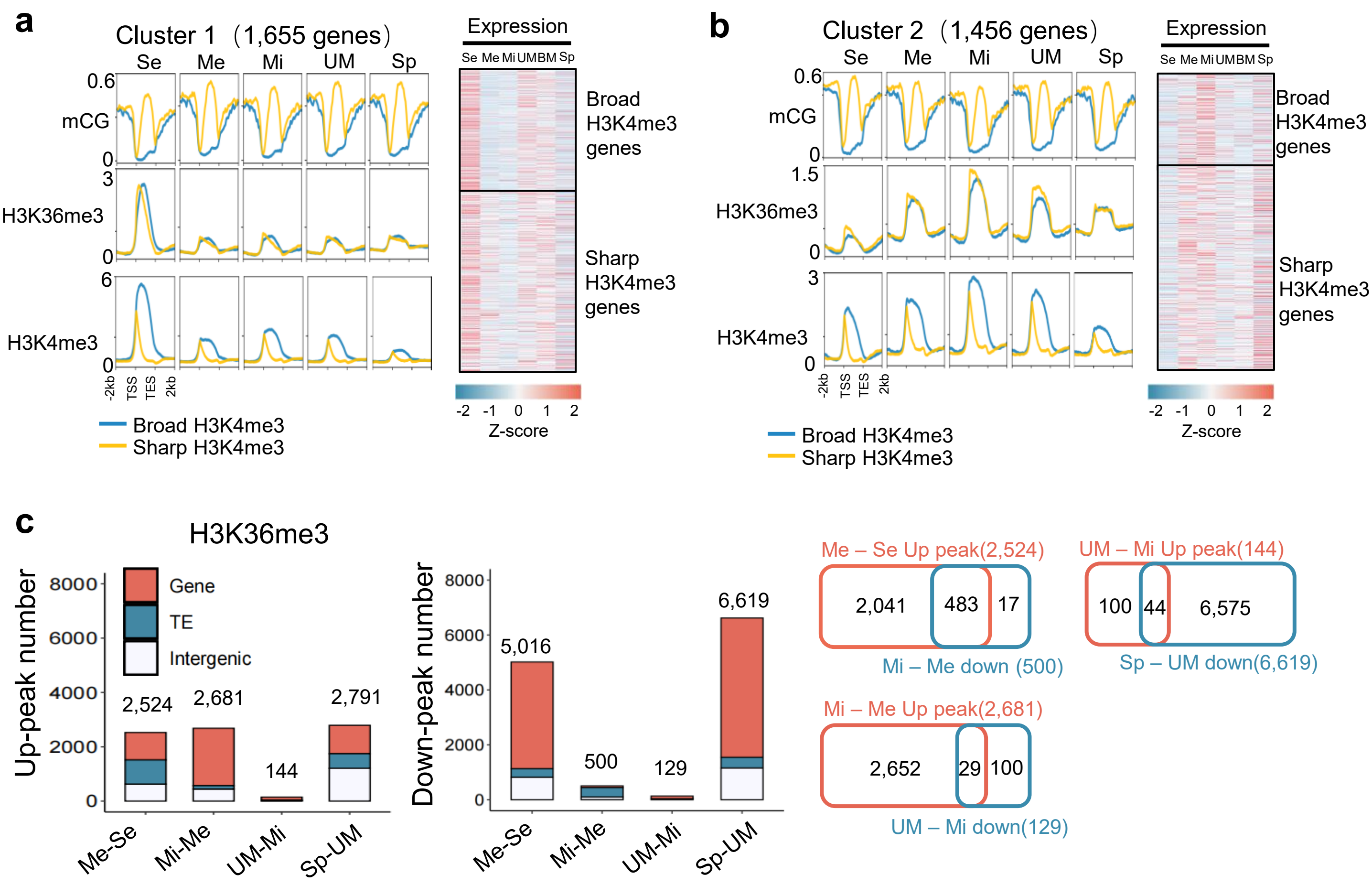

Fig S9. H3K36me3 dynamics during male gamete development.

a and **b**, Clustering by heatmaps of gene H3K36me3 levels in Se, Me and the haploid male cells. Cluster 1: genes with H3K36me3 loss in Me and the haploid male cells; Cluster 2: during male gamete development, genes with increased H3K36me3 at the Me or Mi stages. Cluster 1 and Cluster 2 genes are divided into two groups based on their H3K4me3 distribution profile: group one (upper) with broad H3K4me3 in gene body (with depletion of mCG), group 2 (lower) with sharp H3K4me3 at TSS, enriched with body mCG). **c**, Numbers of differentially methylated peaks between adjacent developmental stages and overlaps between the downregulated peaks in the male cells (relative to the previous stage) with the upregulated ones in the previous stage or cell type.

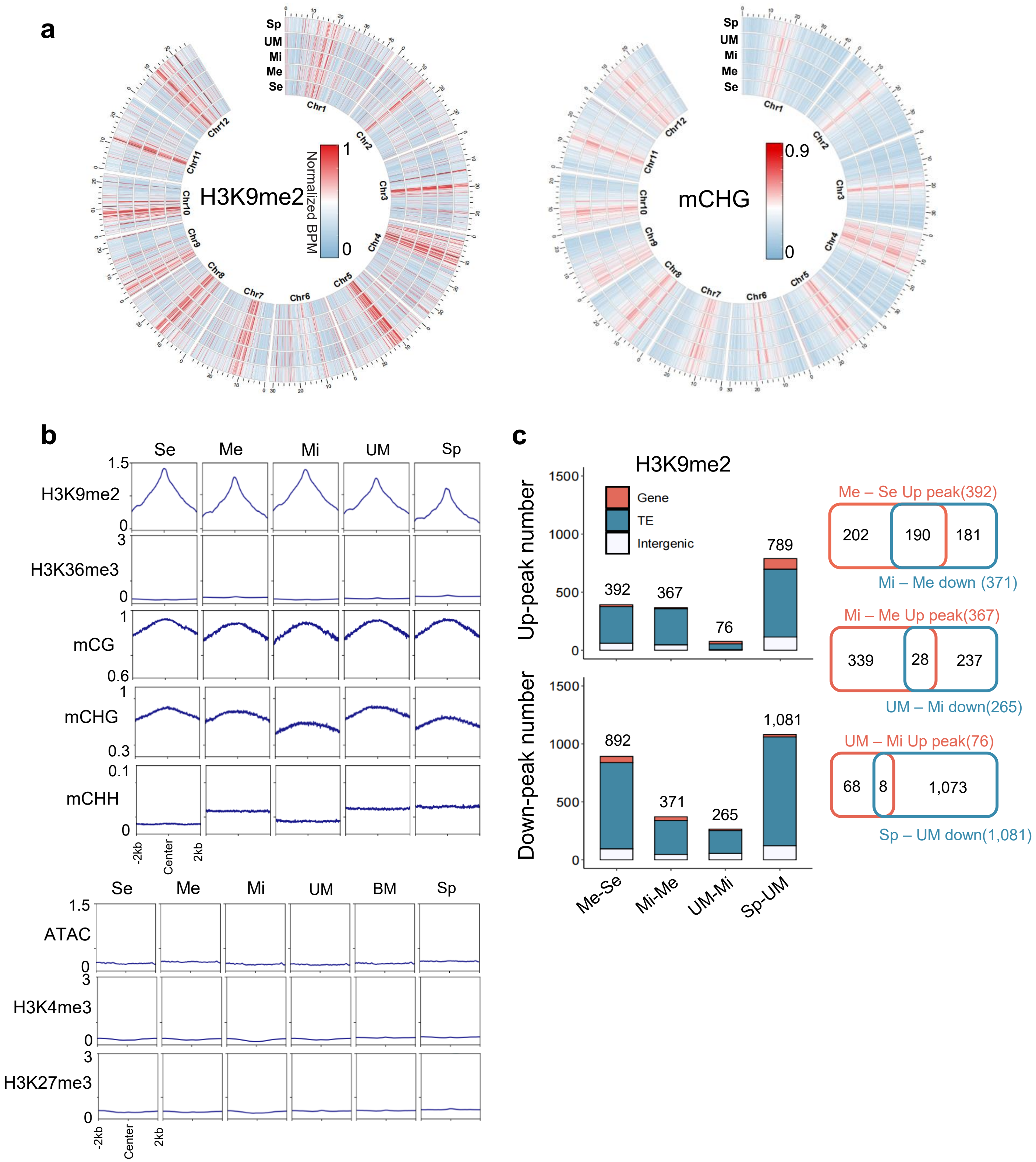

Fig S10. H3K9me2 is relatively stable during male gamete development.

a, Heatmaps showing H3K9me2 and mCHG levels in rice (ZH11) Me and haploid male cells compared with seedling (Se). H3K9me2 and CHG Methylation levels were calculated from 10 kb windows. **b**, Metaplots of H3K9me2 peaks with their ATAC, histone modification, DNA methylation levels in Se, Me and haploid male cells. **c**, Numbers of differentially methylated peaks between adjacent developmental stages and overlaps between the downregulated peaks in the male cells (relative to the previous stage) with the upregulated ones in the previous stage or cell type.

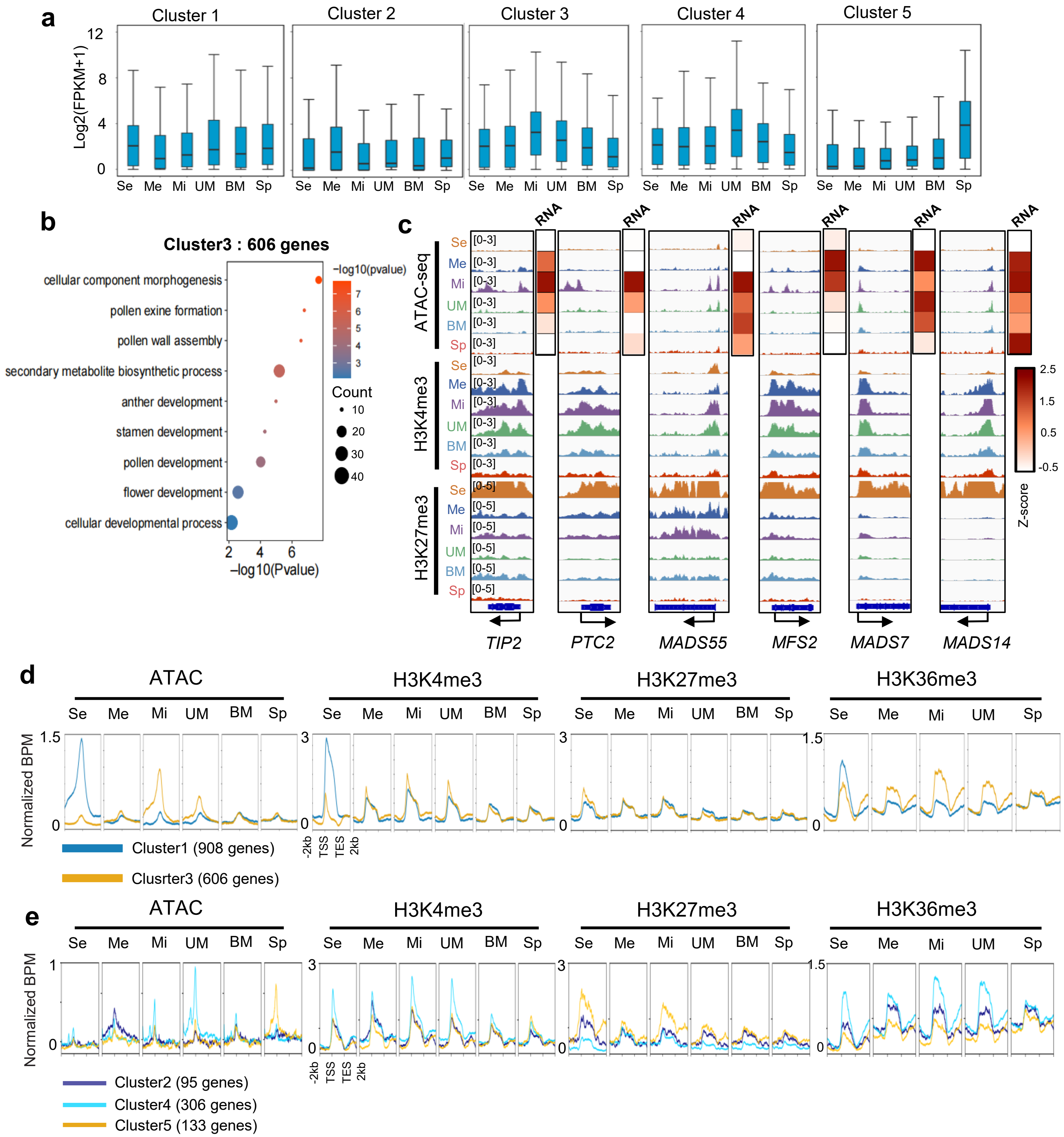

Fig S11. Analysis of genes that gained chromatin accessibility in Me and haploid male cells.

a, Boxplots expression levels of genes corresponding to the cell type specific ATAC clusters (from Fig. 4a) in all cell types for comparison. **b**, GO enrichment analysis of genes of cluster 3 (shown in Fig. 4a). **c**, IGV with histone methylations and expression of representative genes that showed elevated ATAC signals in Me and haploid male cells. **d**, H3K4me3, H3K27me3, H3K36me3 and DNA methylation levels of the genes with Se- (Fig. 4a cluster 1, 908 genes) and Mi-specific (Fig. 4a cluster 3, 606 genes) ATAC signals. **e**, H3K4me3, H3K27me3 and H3K36me3 levels of the genes that gained ATAC signals specifically in Me (from Fig. 4a cluster 2), UM (from Fig. 4a cluster 4), or Sp (from Fig. 4a cluster 5).

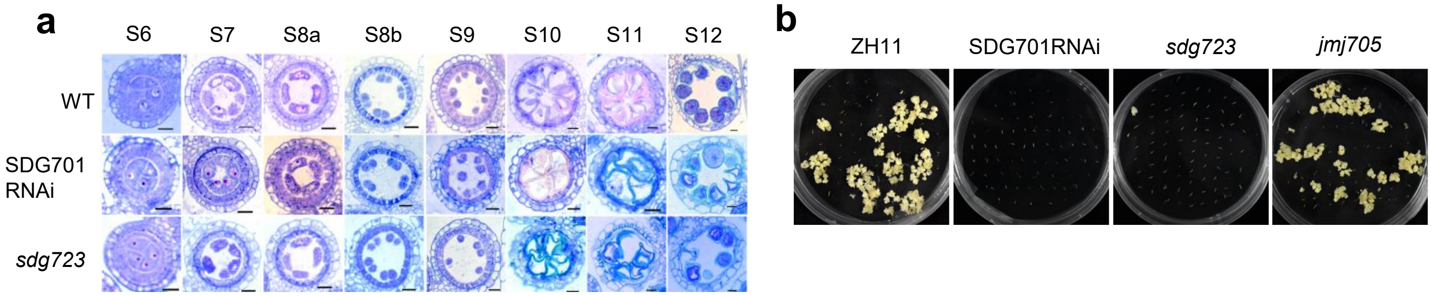

c

Samples	Anthers before induction	Anthers after induction	Induction Rate (%)
ZH11	645	109	16.90
SDG701RNAi	482	0	0.00
sdg723	720	1	0.14
jmj705	619	201	32.47

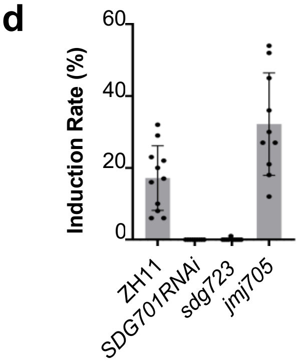

Fig S12. Effects of H3K4me3 methyltransferase gene mutations on microspore development and callus regeneration in *in vitro* culture.

a, Semithin section analysis of anther development in the wild type, SDG701RNAi*, sdg723*. Stage 9 (S9) corresponds to the microspore stage. Bars=20 μm. **b-d**, In vitro culture of anthers at the microspore stage was performed for SDG701RNAi *and sdg723* mutant as well as *jmj705* mutant (H3k27me3 demethylase), and statistical analysis was conducted.

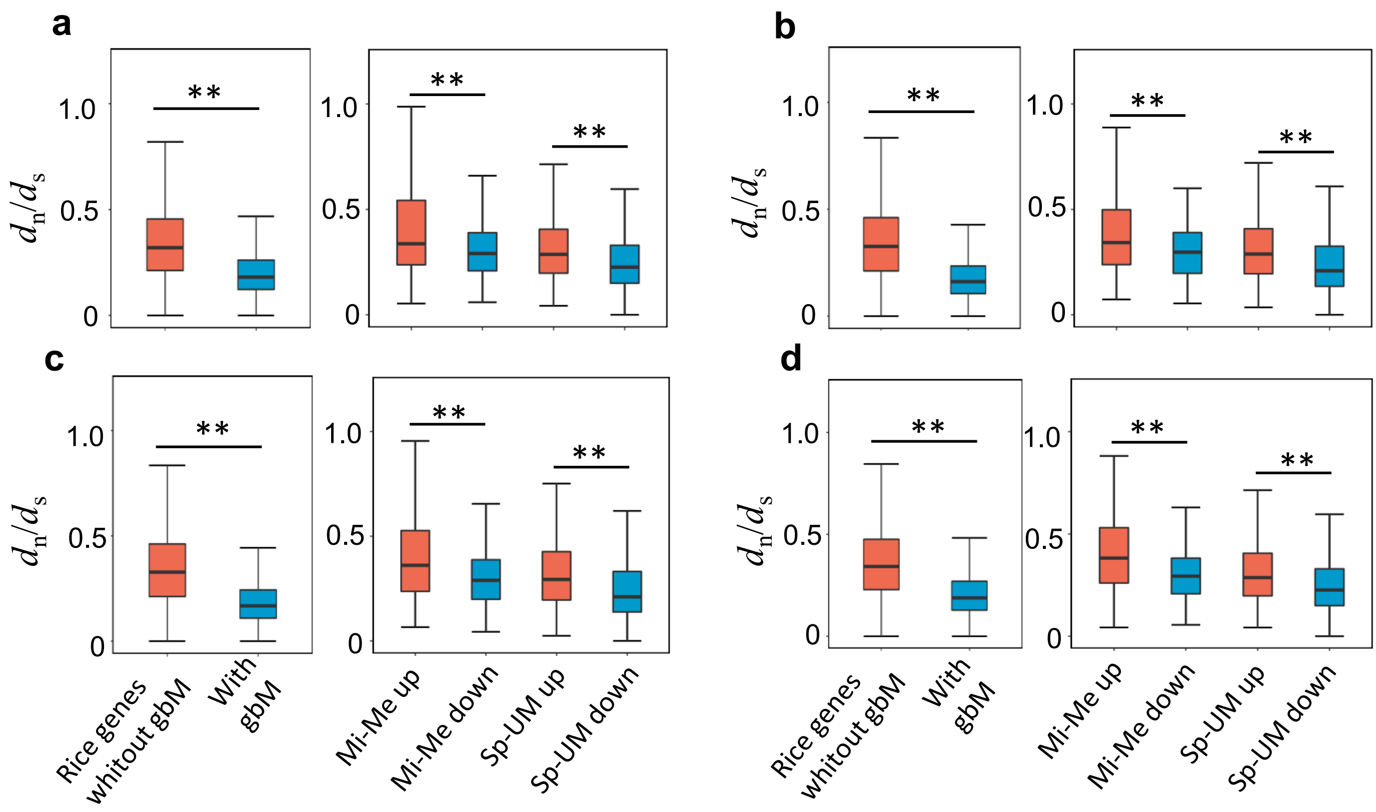

Fig S13. Calculation of d_n/d_s ratios of rice genes with and without gbM.

The dn/ds ratio (the number of nonsynonymous substitutions per nonsynonymous site dn to the number of synonymous substitutions per synonymous site ds) were calculated for total rice genes with gbM (n=8,630) and without gbM (n=19,958), and for genes upregulated (without gbM) and downregulated in Mi versus Me and in Sp versus UM (shown in Figure 5). Calculations were performed with distachyon (**a**), maize (**b**), sorghum (**c**), and wheat (**d**) genes. **, $p < 0.01$, Wilcoxon test adjusted for multiple hypothesis testing with Holm's method.
