# Supplementary material for: Premeiotic chromatin states orchestrate gene expression during male gametogenesis in rice
Source: Genome Biol. 2026 Jun 12;27:192. doi: 10.1186/s13059-026-04129-4 (PMC13262433; doi:10.1186/s13059-026-04129-4)
Supplement: Supplementary file 2 — Additional file 2: Table S1. Rice single male cell type RNA-seq data. Table S2. Rice male cell type histone methylation CUT&Tag data. Table S3. Rice male cell ATAC-seq data. Table S4 Rice male cell BS-seq data. Table S5. TE expressionsof different categories in Se, Me, and haploid male cells. Table S6. H3K4me3 methyltransferase gene mutant microspore CUT&Tag data. [file 13059_2026_4129_MOESM2_ESM.pdf]

Table S1. Rice single male cell type RNA-seq data (100-200 cells)

Cell type	Background	Replicate	clean reads (Millions)	Paired-end uniquely aligned reads (Millions)	Total aligned reads (Millions)	Transcripts with FPKM >1
Seedling	ZH11	1	27.10	21.30 (78.61%)	25.47 (94.00%)	22,069
Seedling	ZH11	2	24.00	18.68 (77.82%)	25.47 (94.03%)	22,220
Seedling	ZH11	3	27.02	21.01 (77.77%)	25.31 (93.68%)	22,042
Meiocyte	ZH11	1	16.41	12.90 (78.65%)	15.69 (95.64%)	20,447
Meiocyte	ZH11	2	14.58	11.69 (80.16%)	13.95 (95.74%)	20,450
Meiocyte	ZH11	3	18.29	14.25 (77.93%)	17.52 (95.58%)	20,475
Tetrad	ZH11	1	16.63	6.91 (41.58%)	10.46 (62.92%)	19,066
Tetrad	ZH11	2	9.50	4.33 (45.56%)	6.68 (70.40%)	19,042
Tetrad	ZH11	3	15.55	6.37 (40.95%)	10.94 (70.61%)	19,068
Microspore	ZH11	1	25.49	17.59 (68.99%)	23.51 (92.25%)	19,797
Microspore	ZH11	2	19.71	13.41 (68.04%)	18.15 (92.10%)	19,694
Microspore	ZH11	3	22.79	15.07 (66.15%)	21.04 (92.36%)	19,714
UM	ZH11	1	18.99	9.55 (50.29%)	17.15 (90.32%)	20,483
UM	ZH11	2	24.08	9.28 (38.55%)	21.47 (89.18%)	20,255
UM	ZH11	3	23.18	7.43 (32.08%)	20.42 (88.13%)	20,133
BM	ZH11	1	22.26	10.89 (48.96%)	20.15 (90.54%)	21,502
BM	ZH11	2	18.59	9.13 (49.14%)	17.01 (91.52%)	21,367
BM	ZH11	3	19.69	9.87 (50.11%)	18.04 (91.63%)	21,489
Sperm	ZH11	1	27.89	18.29 (65.58%)	22.36 (80.02%)	17,689
Sperm	ZH11	2	25.78	17.66 (68.53%)	21.47 (83.30%)	17,652
Microspore	SDG701-RNAi	1	14.15	10.77 (76.15%)	12.06 (85.35%)	18,983
Microspore	SDG701-RNAi	2	11.56	8.32(72.03%)	9.85 (81.60%)	18,747
Microspore	SDG701-RNAi	3	10.23	73.69 (72.00%)	8.39 (82.04%)	18,780
Microspore	*sdg723*	1	10.45	7.90 (75.65%)	8.78 (84.05%)	18,119
Microspore	*sdg723*	2	11.07	9.01 (81.39%)	9.77 (88.34%)	18,028
Microspore	*sdg723*	3	14.05	11.37 (80.92%)	12.35 (87.97%)	18,112

Table S2. Rice male cell type histone methylation CUT&Tag data (with seedling cells as somatic controls)

Cell type	Background	Replicate	Antibody	clean reads (Millions)	Total aligned reads (Millions)	Peak Number
Seedling	ZH11	1	H3K4me3	43.59	43.07 (98.83%)	29,175
Seedling	ZH11	2	H3K4me3	34.75	32.57 (93.74%)	29,887
Meiocyte	ZH11	1	H3K4me3	11.74	11.34 (96.65%)	35,336
Meiocyte	ZH11	2	H3K4me3	10.63	10.26 (96.55%)	34,876
Microspore	ZH11	1	H3K4me3	21.96	21.33 (97.15%)	33,984
Microspore	ZH11	2	H3K4me3	23.30	22.76 (97.71%)	33,284
UM	ZH11	1	H3K4me3	16.20	15.78 (97.46%)	32,120
UM	ZH11	2	H3K4me3	17.71	17.24 (97.39%)	32,971
BM	ZH11	1	H3K4me3	12.43	11.93 (95.98%)	31,423
BM	ZH11	2	H3K4me3	14.51	13.97 (96.28%)	30,767
Sperm	ZH11	1	H3K4me3	14.18	13.68 (96.51%)	28,036
Sperm	ZH11	2	H3K4me3	13.34	12.87 (96.49%)	27,636
Seedling	ZH11	1	H3K9me2	19.30	18.87 (97.79%)	8,733
Seedling	ZH11	2	H3K9me2	20.75	20.30 (97.87%)	8,558
Meiocyte	ZH11	1	H3K9me2	15.95	14.60 (91.58%)	8,885
Meiocyte	ZH11	2	H3K9me2	14.10	12.84 (91.08%)	9,534
Microspore	ZH11	1	H3K9me2	12.11	10.47 (86.46%)	9,292
Microspore	ZH11	2	H3K9me2	16.65	14.59 (87.67%)	9,084
UM	ZH11	1	H3K9me2	17.85	15.75 (88.25%)	8,935
UM	ZH11	2	H3K9me2	15.75	15.07 (95.72%)	8,456
Sperm	ZH11	1	H3K9me2	11.52	7.70 (66.91%)	10,741
Sperm	ZH11	2	H3K9me2	11.94	8.05 (67.43%)	11,141
Seedling	ZH11	1	H3K27me3	29.40	28.87 (98.23%)	1,0203
Seedling	ZH11	2	H3K27me3	26.33	25.86 (98.25%)	1,0731
Meiocyte	ZH11	1	H3K27me3	19.50	17.97 (92.16%)	14,315
Meiocyte	ZH11	2	H3K27me3	19.86	19.08 (92.10%)	14,456
Microspore	ZH11	1	H3K27me3	15.49	15.07 (97.43%)	13,370
Microspore	ZH11	2	H3K27me3	19.76	19.22 (97.29%)	12329
UM	ZH11	1	H3K27me3	43.25	34.54 (79.88%)	10,824
UM	ZH11	2	H3K27me3	12.84	7.29 (56.84%)	9,789
BM	ZH11	1	H3K27me3	35.21	33.42 (94.93%)	11,819
BM	ZH11	2	H3K27me3	31.52	26.56 (85.02%)	10,935
Sperm	ZH11	1	H3K27me3	73.81	52.93 (71.72%)	7,321
Sperm	ZH11	2	H3K27me3	45.37	32.40 (71.42%)	6,879
Seedling	ZH11	1	H3K36me3	39.58	39.26 (99.20%)	14,176
Seedling	ZH11	2	H3K36me3	33.11	32.83 (99.17%)	13,668
Meiocyte	ZH11	1	H3K36me3	40.17	39.36 (97.99%)	15,876
Meiocyte	ZH11	2	H3K36me3	49.73	48.21 (96.96%)	16,121
Microspore	ZH11	1	H3K36me3	37.09	36.64 (98.79%)	14,781
Microspore	ZH11	2	H3K36me3	36.97	36.35 (98.34%)	15,121
UM	ZH11	1	H3K36me3	47.49	46.76 (98.48%)	13,671
UM	ZH11	2	H3K36me3	42.33	41.66 (98.42%)	13,345
Sperm	ZH11	1	H3K36me3	34.78	33.28 (95.69%)	17,781
Sperm	ZH11	2	H3K36me3	71.25	68.15 (95.66%)	18,987
Meiocyte	ZH11	1	H3	38.21	33.51 (87.71%)	-
Microspore	ZH11	1	H3	25.39	23.59 (92.92%)	-
UM	ZH11	1	H3	34.26	32.10 (93.70%)	-
BM	ZH11	1	H3	34.23	31.94 (93.32%)	-
Sperm	ZH11	1	H3	38.02	32.03 (84.25%)	-
Seedling	ZH11	1	H3	32.79	32.29 (98.49%)	-

Table S3. Rice male cell ATAC-seq data

Cell type	Background	Replicate	clean reads (Millions)	Total aligned reads (Millions)
Seedling	NIP	1	18.49	17.80 (96.28%)
Seedling	NIP	2	56.72	54.72 (96.48%)
Meiocyte	ZH11	1	22.06	20.24 (91.75%)
Meiocyte	ZH11	2	21.47	19.73 (91.91%)
Microspore	ZH11	1	24.67	22.53 (91.36%)
Microspore	ZH11	2	18.26	16.66 (91.28%)
UM	ZH11	1	9.6	8.93 (93.03%)
UM	ZH11	2	17.08	15.89 (93.09%)
BM	ZH11	1	14.79	12.89 (87.17%)
BM	ZH11	2	14.57	12.72 (87.36%)
Sperm	ZH11	1	11.11	10.23 (92.11%)
Sperm	ZH11	2	8.9	8.19 (92.12%)

Table S4. Rice male cell BS-seq data

Cell Type	Background	Replicate	Mapping reads （paired-end） (Millions)	Bisulfite conversion rate §	Mapping reads coverage depth (X)
Seedling	ZH11	1	36.72	0.985	16.29
Seedling	ZH11	2	34.81	0.992	15.60
Meiocyte	ZH11	1	23.74	0.971	15.61
Meiocyte	ZH11	2	23.09	0.984	15.19
Microspore	ZH11	1	9.28	0.990	6.10
Microspore	ZH11	2	12.14	0.974	7.98
UM	ZH11	1	25.33	0.969	16.66
UM	ZH11	2	19.67	0.985	12.94
Sperm	ZH11	1	15.25	0.989	10.03
Sperm	ZH11	2	17.09	0.991	11.24

§ , Bisulfite conversion rate was calculated by subtracting chloroplast DNA methylation ratio.

Table S5. TE expressions (Reads per million mapped reads, RPM>1) of different categories in Se, Me, and haploid male cells.

	Seedlings		Meiocyte		Microspore		Unicellular Microspore		Bicellular Microspore		Sperm	
TE_Type	TE expression counts	TE expression proportion of annotation	TE expression counts	TE expression proportion of annotation	TE expression counts	TE expression proportion of annotation	TE expression counts	TE expression proportion of annotation	TE expression counts	TE expression proportion of annotation	TE expression counts	TE expression proportion of annotation
Dna/Dta	543	8.13%	503	7.53%	506	7.58%	578	8.65%	553	8.28%	547	8.19%
Dna/Dtc	990	4.88%	971	4.79%	1008	4.97%	1346	6.64%	1159	5.72%	1031	5.09%
Dna/Dth	560	6.44%	520	5.98%	555	6.38%	570	6.55%	572	6.57%	568	6.53%
Dna/Dtm	3934	9.12%	2997	6.95%	3310	7.68%	4971	11.53%	3840	8.90%	3063	7.10%
Dna/Dtt	6026	5.34%	5514	4.89%	5883	5.22%	7502	6.65%	6397	5.67%	5786	5.13%
Dna/Helitron	9116	7.15%	8102	6.35%	8058	6.32%	9233	7.24%	8954	7.02%	8396	6.58%
Ltr/Copia	659	3.60%	642	3.51%	626	3.42%	1406	7.68%	899	4.91%	727	3.97%
Ltr/Gypsy	1485	2.59%	2022	3.52%	2585	4.50%	6113	10.64%	3249	5.66%	2612	4.55%
Ltr/Unknown	15	0.94%	12	0.75%	12	0.75%	39	2.45%	18	1.13%	22	1.38%
Mite/Dta	308	5.53%	222	3.98%	253	4.54%	230	4.13%	248	4.45%	259	4.65%
Mite/Dtc	21	3.88%	20	3.70%	17	3.14%	25	4.62%	23	4.25%	14	2.59%
Mite/Dth	321	4.71%	309	4.54%	330	4.85%	421	6.18%	324	4.76%	329	4.83%
Mite/Dtm	602	4.56%	461	3.49%	638	4.83%	1476	11.18%	769	5.82%	473	3.58%
Mite/Dtt	750	6.36%	539	4.57%	557	4.72%	978	8.29%	657	5.57%	484	4.10%
Sine	148	3.76%	137	3.48%	157	3.99%	124	3.15%	260	6.60%	211	5.36%

Table S6. H3K4me3 methyltransferase gene mutant microspore CUT&Tag data

Cell type	Background	Replicate	Antibody	clean reads (Millions)	Total aligned reads (Millions)
Meiocyte	SDG701-RNAi	1	H3K4me3	23.64	23.26 (98.41%)
Meiocyte	SDG701-RNAi	2	H3K4me3	17.07	16.76 (98.18%)
Meiocyte	*sdg723*	1	H3K4me3	28.54	27.63 (96.83%)
Meiocyte	*sdg723*	2	H3K4me3	31.65	30.64 (96.82%)
Microspore	SDG701-RNAi	1	H3K4me3	28.43	28.06 (98.71%)
Microspore	SDG701-RNAi	2	H3K4me3	15.72	15.48 (98.53%)
Microspore	*sdg723*	1	H3K4me3	14.17	13.80 (97.45%)
Microspore	*sdg723*	2	H3K4me3	12.05	11.75 (97.58%)
